# A global parametric rain model for landfalling tropical cyclones: a case study for the U.S.

**DOI:** 10.1007/s11069-026-08150-5

**Published:** 2026-04-29

**Authors:** King Heng Lau, Sacha Czernichow, Nathan Sparks, Ralf Toumi

**Affiliations:** 1 Department of Physics, Imperial College London, London, SW7 2AZ UK; 2 Université Paris-Saclay, ENS Paris-Saclay, 91190 Gif-sur-Yvette, France

**Keywords:** Tropical cyclone, Tropical cyclone precipitation, Climate change, Flood risk

## Abstract

Rainfall associated with tropical cyclones (TCs) is a crucial driver of TC hazards, yet estimating TC rain risk from observations is hindered by their relative infrequency. Parametric TC rain models coupled with stochastic TC risk models provide an efficient mean for quantifying such risk. This study introduces a new parametric rain model for landfalling TCs, integrated into the Imperial College Storm Model (IRIS), a statistical-thermodynamic global TC hazard model. Using a 10,000-year simulation, IRIS reproduces observed global return periods of landfall rain rate, storm-total rain volume, and lifetime rain production over land. Over the United States, the model captures observed rainfall climatology and event accumulations with skill comparable to or slightly exceeding existing parametric models. The capability of the model for climate projection is demonstrated through a United States case study using a storyline approach that isolates thermodynamic effects, specifically increases in potential intensity and total column water, under a +2 °C global warming scenario. The pre-landfall maximum azimuthal mean rain rate of United States hurricanes increases by 20.1%, while contraction of the rain field limits the storm-total rain volume increase to 3.9%. Across their lifetimes over land, hurricanes produce 14.6% more rainfall. Spatially, warming enhances rain rates and accumulations across the eastern and southern United States, with the largest absolute increases in the southeast but the strongest relative increases inland and in the northeast, indicating greater inland and poleward penetration of hurricane rainfall under warming.

**Supplementary Information:**

The online version contains supplementary material available at 10.1007/s11069-026-08150-5.

## Introduction

Tropical cyclones (TCs) produce intense and widespread rainfall that often leads to catastrophic flooding, landslides, and mudslides, posing severe risks to both coastal and inland communities. Globally, TC-induced rainfall has been identified as a major driver of fatalities and economic losses, often exceeding those caused by wind. In the United States (U.S.), for example, nearly one-quarter of all deaths directly attributed to Atlantic TCs between 1963 and 2012 were due to rain-induced flooding (Rappaport 2014), while in China and the Caribbean, TC rainfall has often been the primary cause of economic losses (Wen et al. 2018; Collalti and Strobl 2022). Similar findings in South Korea highlight rainfall as the most decisive factor in determining overall TC impacts (Park et al. 2015; Nam et al. 2018). As climate change is projected to intensify TC rainfall (Knutson et al. 2020), the accurate representation of rainfall in TC risk assessments is becoming increasingly critical for improving damage projections and informing adaptation strategies.

A multitude of methods have been employed to project past and future changes in TC rainfall. Recent studies based on satellite estimates of TC rain found a global increase in overall TC rainfall due to an increase in rain rate in the TC outer region (Tu et al. 2021; Guzman and Jiang 2021). This increasing trend is expected to continue under global warming, as the capacity of the air to hold water vapour increases. The thermodynamic expectation of this increase is ~7% °C^−1^ of sea surface temperature (SST) warming, as governed by the Clausius–Clapeyron (C–C) relation. Projections from climate model studies indicate that the near-storm rainfall rates are projected to increase globally by an average of 14% for a global mean warming of +2 °C (Knutson et al. 2020), with increases generally matching or slightly exceeding the C–C increase. While some studies used global climate models (GCMs) with relatively coarser resolutions (Wehner et al. 2015; Yoshida et al. 2017; Villarini et al. 2014; Kim et al. 2014; Yamada et al. 2017; Stansfield et al. 2020), others employed dynamical downscaling using higher-resolution regional climate models (RCMs) (Knutson et al. 2013; Wright et al. 2015; Knutson et al. 2015; Tsuboki et al. 2015; Hill and Lackmann 2011; Knutson et al. 2022) to provide regional and global projections. Although most studies have examined basin-wide rainfall rates, some have focused specifically on TC rainfall during and after landfall (Wright et al. 2015; Liu et al. 2019; Scoccimarro et al. 2014; Knutson et al. 2022), which represents the most critical phase from a risk perspective as TCs directly impact communities.

The use of GCMs and RCMs to provide projections of TC rain comes with key limitations. GCMs lack the spatial resolution needed to realistically capture mesoscale TC structures, while high-resolution RCMs, though better at representing storm dynamics and rainfall, are computationally demanding and rely on GCM outputs for long-term climatologies. These constraints make it challenging to simulate large ensembles of storms over extended periods. Parametric TC rain models have been developed to provide an efficient alternative to model TC rainfall. Statistical models such as the Rainfall Climatology and Persistence model (R-CLIPER) (Tuleya et al. 2007), based on the work of Lonfat et al. (2004) using TRMM-derived climatologies, estimate rainfall from storm intensity, size, and translation speed but assume symmetric structures, neglecting shear and topography. The Parametric Hurricane Rainfall Model (PHRaM) (Lonfat et al. 2007) extends R-CLIPER by incorporating parametrisations for vertical wind shear and orographic effects, improving spatial realism. The Parametric Hurricane Rainfall Model with Moisture (PHRaMM) (Kim et al. 2022) added total column water (TCW) to the existing PHRaM framework as a predictor of the symmetric TC rain field to incorporate the effect of changing TCW on TC rainfall. Physically based models include the modified Smith for rainfall model (MSR) (Langousis and Veneziano 2009), which links rainfall to boundary-layer convergence and storm motion over open water, and the physics-based TC rainfall model (TCR) (Zhu et al. 2013; Lu et al. 2018), which estimates rainfall from vertical vapour fluxes influenced by frictional convergence, shear, topography, and storm evolution.

Parametric TC rain models have been employed to assess TC rainfall risk. Zhu et al. (2013) applied the TCR model to simulate rainfall from 3,085 synthetic TCs generated from a global synthetic dataset (Emanuel 2006) passing within 100 km of the Texas. The resulting rainfall climatology showed good agreement with rain gauge observations at daily and event timescales. Emanuel (2017) extended this approach to simulate TC rainfall for synthetic storms near Texas under a future climate (2081–2100) following the Intergovernmental Panel on Climate Change AR5 representative concentration pathway (RCP) 8.5, using projections from six climate models. He estimated an 18-fold increase in the annual probability of areally averaged hurricane rainfall comparable to Hurricane Harvey. Gori et al. (2022) followed the same approach to downscale TCs from eight Coupled Model Intercomparison Project phase 6 (CMIP6) GCMs under the SSP5-8.5 scenario and project a 32% increase in inner core mean TC rain rate. Kim et al. (2022) used the PHRaMM model to assess the impacts of TC intensity and environmental moisture increase on TC rainfall under a future climate. Rather than simulating synthetic storms, they evaluated the sensitivity of average TC rain rates to 1%–10% increases in storm intensity and a 14% increase in TCW for 288 TCs that made landfall in the western North Pacific (WNP) basin during 1998–2014. They found super C–C scaling for storm-average rainfall (17.96–20.91%) but sub C–C scaling for inner-core rainfall (4.61–8.51%).

Recently, Sparks and Toumi (2024) introduced the Imperial College Storm Model (IRIS), a statistical-thermodynamic global TC hazard model built within a novel stochastic framework. Unlike previous stochastic TC models that simulate storms from genesis (Vickery et al. 2000; James and Mason 2005; Lee et al. 2018; Bloemendaal et al. 2020; Emanuel 2006; Lin et al. 2023), IRIS focuses on the critical decay phase, recognising that landfall wind speed primarily depends on the location and value of the lifetime maximum intensity (LMI) (Wang and Toumi 2022). By reducing the problem to modelling TC decay, IRIS avoids the error accumulation typical of full life cycle simulations and enables the realistic representation of related quantities such as storm size and the pressure–wind relationship during this phase. Each synthetic TC track is generated by perturbing observed “parent” tracks, with stochastic LMIs derived from the thermodynamically constrained potential intensity (PI) field (Emanuel 1986). Landfall intensity is then obtained through a stochastic decay model applied along each perturbed track. IRIS can reproduce key statistical properties of observed TCs and globally realistic return periods of landfall wind speed (Sparks and Toumi 2024). By prescribing historical and projected changes in PI, IRIS has been used for climate change attribution of TC landfall intensity (Clarke et al. 2025; Sparks and Toumi 2025a) and for quantifying both current (Toumi and Sparks 2025) and future (Sparks and Toumi 2025b) TC wind risk.

In this study, a new parametric TC rain model for IRIS is proposed to simulate TC rainfall at and after landfall. By integrating the rain model into IRIS, we demonstrate that the framework can be used to quantify future TC rainfall risk under climate change. The paper is organised as follows: Sect. 2 describes the datasets used for model development, the formulation of the proposed parametric rain model, and the storyline approach adopted to assess future TC rainfall risk. In Sect. 3, the performance of the rain model is evaluated, followed by a case study assessing future U.S. hurricane rainfall risk. Section  4 discusses the key findings, and the main conclusions are summarised in Sect. 5.

## Data and methodology

### Datasets

#### International best track archive for climate stewardship (IBTrACS) version 4r01

The International Best Track Archive for Climate Stewardship (IBTrACS) Version 4r01 serves as the source of best track TC data and for selecting TC cases used to train the parametric rainfall models. The time period spans from 1 January 1998 to 31 December 2023 (hereafter the “analysis period”). Within this 26-year timeframe, all TC instances in IBTrACS meeting the following criteria were included:

- The system was classified as “Tropical” by the reporting agency, as indicated by the “NATURE” variable in IBTrACS. This ensures that only TC systems are considered, excluding non-tropical systems such as extratropical cyclones. This approach is commonly followed in existing TC studies (e.g., Tu et al. (2021, 2022); Guzman and Jiang (2023); Zhong et al. (2026)).
- The system attained a minimum intensity of tropical storm (TS) on the Saffir–Simpson Hurricane Scale (SSHS), as indicated by the “USA_SSHS” variable in IBTrACS. This threshold was chosen as IRIS does not simulate decay below the TS intensity level.
- The TC centre was located within the latitudinal band of 45 °S–45 °N, based on the “USA_LAT” variable in IBTrACS. This follows the approach of previous global TC studies (e.g., Tu et al. (2021, 2022)) and reduces the likelihood of including extratropical systems.
- The track type (“TRACK_TYPE” in IBTrACS) was designated as the main track (“MAIN”), with spurs (“spur”) excluded. Spurs are short-lived track segments that arise from positional uncertainty due to observational limitations (Knapp 2024); excluding them prevents double counting of the same system. This approach is widely adopted in TC studies (e.g., Guzman and Jiang (2023); Zhong et al. (2026)).

After the filtering process, a total of 2,214 TC cases and 76,700 instantaneous records were obtained.

From IBTrACS, the maximum sustained wind speed of each TC instance ($V_{max}$; “USA_WIND” in IBTrACS) and the quadrant-averaged radius of gale-force wind ($R_{18}$; “USA_R34” in IBTrACS) reported by the relevant US agency were extracted for fitting the proposed parametric rain model (Sects. 2.3–2.4).

#### Integrated multi-satellite retrievals for GPM (IMERG) V07

The Integrated Multi-satellitE Retrievals for GPM (IMERG) (Huffman et al. 2023) is a unified algorithm that produces global precipitation estimates by combining observations from the Global Precipitation Measurement (GPM) satellite constellation, an international network of satellites. In this study, the gauge-calibrated multi-satellite precipitation product (Final) (*precipitation*) is employed. The generation of *precipitation* involves intercalibrating, merging, and interpolating all available microwave-based satellite precipitation estimates, supplemented by microwave-calibrated infrared observations, precipitation gauge analyses, and, where applicable, additional estimators. The resulting product provides high-resolution precipitation data, available globally at 0.1° spatial resolution, spanning the period from January 1998 to the present. Precipitation rates are reported in units of mm h^−1^.

From IMERG, the TC rain fields of selected TC instances were extracted for periods before and after landfall. The TC rain field is defined as the region extending up to 500 km from the storm centre, consistent with definitions adopted in several previous studies (Lavender and Mcbride 2021; Lin et al. 2015; Kamahori 2012; Matyas 2014; Lonfat et al. 2004; Tuleya et al. 2007; Kim et al. 2022; Lonfat et al. 2007). Grid points were selected based on their great-circle distance from the TC centre, ensuring that the 500 km radius is defined in physical distance rather than grid spacing despite the latitude-dependent resolution of the IMERG grid.

#### ECMWF reanalysis v5 (ERA5)

The European centre for medium-range weather forecasts reanalysis version 5 (ERA5) dataset (Hersbach et al. 2020) represents the fifth-generation atmospheric reanalysis of the global climate produced by ECMWF through the Copernicus Climate change service (C3S). ERA5 provides global estimates of a wide range of atmospheric, land, and oceanic variables by integrating diverse observational datasets, including satellite and in-situ measurements, with advanced modelling and data assimilation techniques. The dataset offers global coverage on a 31-km grid and resolves the atmosphere across 137 vertical levels, extending from the surface up to 80 km. ERA5 spans the period from 1940 to the present, with data provided at an hourly resolution. ERA5-Land (Sabater and 2019) is a replay of the land component of the main ERA5 reanalysis, offering finer spatial resolution (9 km) for land surface variables over land.

In this study, TCW was extracted from ERA5 monthly averaged data on single levels (Hersbach et al. 2023), while the (invariant) surface geopotential height (*GH*) was derived from ERA5-Land hourly data (Sabater and 2019), for use in fitting the parametric rain model (Sects. 2.3–2.4). In line with the definition of the TC rain field (Sect. 2.1.2) and previous study (Kim et al. 2022), the mean value of each variable within 500 km of the TC centre was used, with grid cells included based on their great-circle distance from the storm centre. Despite the differences in spatial resolution between ERA5 and ERA5-Land, the temporal and spatial averaging employed means that the effective spatial scale of the predictors is substantially larger than the native grid spacing of either dataset, thus the impact of resolution mismatch should be minimal.

In addition, monthly averaged SST and vertical profiles of temperature and humidity were obtained from ERA5 monthly averaged data on single levels (Hersbach et al. 2023) for calculating the monthly mean PI for 1979–2023 using an algorithm (Bister and Emanuel 2002; Gilford 2021), following Sparks and Toumi (2025b). The monthly mean TCW was also obtained for the same period. The temporal trends of PI and TCW were then computed for each month, which were used to drive the pre-industrial, present, and future climate scenarios in IRIS (Supplementary Text S1).

#### Natural earth

Natural Earth vector data was used to identify land and country boundaries relevant to TC landfall detection. Natural Earth is a publicly available, cartographically optimised dataset that provides global physical and cultural features at multiple map scales. For this study, the 1:50 million resolution “Land” (physical vectors) and “Admin 0 – Countries” (cultural vectors) layers were used, which offer generalised yet accurate representations of coastlines and national boundaries suitable for our analysis. Following the methodology for computing land variables in IBTrACS (Knapp 2024), islands smaller than 1400 km^2^ were removed from the Natural Earth land polygons as they are assumed to have less impact on the TC structure. In addition to detecting TC landfall, land polygons were used to estimate the fraction of land surrounding a TC (*LandFrac*), defined as the proportion of land area within 500 km of the TC centre, for use in fitting the post-landfall rainfall model (Sect. 2.4).

### Defining a landfall episode

In this study, a TC was classified as either over land or over ocean, depending on whether its centre lies within one of the Natural Earth land polygons. A TC landfall episode begins at the last ocean point before landfall and ends at the final land point preceding either (a) the decay of the TC below TS strength (< 1.75 ms^−1^) over land, or (b) the re-entry of the TC into the ocean. Only landfalls with a last-ocean-point intensity exceeding Category 1 (CAT1+) on the SSHS (≥ 33 ms^−1^) were included in this study. This restriction was adopted because TSs and CAT1+ TCs exhibit different structural and dynamical characteristics, such as the presence of a well-defined eyewall and more organised convection, which lead to distinct rainfall distributions and scaling relationships. Restricting the analysis to CAT1+ TCs therefore provides a more physically consistent basis for fitting the proposed rainfall model. The country of landfall was determined by checking the intersection of the line segment connecting the last ocean point and the first land point with the Natural Earth country polygons (Sect. 2.1.4). Once the intersection point was identified, the time of landfall was estimated by linearly interpolating the TC position along this line segment, assuming a constant translation speed.

### Pre-landfall rain model

To represent the symmetric TC rain field at the last ocean location prior to landfall, a pre-landfall parametric rain model was developed and implemented in IRIS. The formulation follows the R-CLIPER approach (Tuleya et al. 2007), which expresses the TC rain rate (*P*) as a function of radius (*r*):

1 $$\begin{aligned} P(r)&= P_0 + (P_m - P_0)\left( \frac{r}{R_m}\right) , \qquad&r&< R_m \end{aligned}$$

2 $$\begin{aligned} P(r)&= P_m \exp \!\left[ -\frac{(r-R_m)}{R_e}\right] , \qquad&r&\ge R_m \end{aligned}$$

where $P_0$ is the rain rate at the storm centre ($r=0$), and $P_m$ is the maximum rain rate occurring at $r=R_m$. According to this formulation, rainfall increases linearly from $P_0$ at the centre to $P_m$ at radius $R_m$, and then decays exponentially beyond $R_m$ with an *e*-folding length scale of $R_e$. The four rainfall parameters were calculated for all global last ocean points of CAT1+ landfall episodes. Over the analysis period, a total of 483 CAT1+ landfall episodes (and thus 483 last ocean points) were identified worldwide, corresponding to 383 unique TC cases. An illustration of parameter calculation is given in Supplementary Fig. S1.

In R-CLIPER, *P* is predicted solely by $V_{max}$ (Tuleya et al. 2007). More recently, a modified version with TCW as an additional predictor has been proposed (Kim et al. 2022). The four parameters, $P_m$, $R_m$, $P_0$, and $R_e$, are then predicted by a linear regression equation. In this study, we follow the same linear regression approach, but with the following equations:

3 $$\begin{aligned} \ln {P_m}&= a_1 + b_1\ln {V_{max}} + c_1\ln {TCW} + \epsilon _1 \qquad&\epsilon _1&\sim \mathcal {N}(\cdot ) \end{aligned}$$

4 $$\begin{aligned} \ln {R_m}&= a_2 + b_2P_m + \epsilon _2 \qquad&\epsilon _2&\sim \mathcal {B}(\cdot ) \end{aligned}$$

5 $$\begin{aligned} \ln {P_0}&= a_3 + b_3\frac{P_m}{R_m} + \epsilon _3 \qquad&\epsilon _3&\sim \mathcal {W}_\textrm{min}(\cdot ) \end{aligned}$$

6 $$\begin{aligned} \ln {R_e}&= a_4 + b_4P_m + c_4R_{18} + \epsilon _4 \qquad&\epsilon _4&\sim \mathcal {B}(\cdot ) \end{aligned}$$

where *a*, *b*, and *c* are the regression coefficients, and $\epsilon$ is the noise term drawn from a distribution, representing variability in the dependent variable that is not explained by the predictors included in the model. In this context, $\epsilon$ can be interpreted as the aggregate effect of unresolved or unmodelled physical processes (e.g. environmental wind shear, dry-air intrusions, and variations in TC vertical structure), as well as measurement uncertainty and intrinsic stochastic variability. $\mathcal {N}$, $\mathcal {B}$, and $\mathcal {W}_\textrm{min}$ represent the normal, beta, and Weibull Minimum Extreme Value distributions, respectively.

Eqs. 3, 4, 5, and 6 were fitted globally to the last ocean points of CAT1+ landfall episodes using least-squares regression. Each predictor was standardised by its observed mean and standard deviation, as summarised in Supplementary Table S1. The resultant regression coefficients and the coefficient of determination ($R^2$) are presented in Table 1. The partial regression coefficients are significant at the 95% confidence level. A 5-fold, leave-one-out cross-validation was carried out to evaluate the robustness of the estimated coefficients. The standard errors of the coefficients from the cross-validation were all significantly smaller (< 1%) than the coefficients in magnitude, which demonstrates the robustness of the regression results. Additionally, all noise distributions passed the two-sample Kolmogorov-Smirnov (KS) test for goodness of fit at 5% significance level (Supplementary Fig. S2). The fitted parameters for the noise distributions are provided in Supplementary Table S2.

**Table 1:** Estimated regression coefficients for the parameters of the pre-landfall rain model defined in Eqs. 3, 4, 5, and 6. Reported are the regression coefficients (*a*, *b*, *c*), their standard errors obtained from a 5-fold leave-one-out cross-validation (in parentheses), and the pooled $R^2$ values.

	*a*	*b*	*c*	Pooled $R^2$
* P*_*m*_ (mm h^−1^)	*a*_1_ = 2.45 (0.00384)	*b*_1_ = 0.220 (0.00944)	*c*_1_ = 0.159 (0.00916)	0.224
* R*_*m*_ (km)	*a*_2_ = 3.68 (0.0102)	*b*_2_ = 0.350 (0.0174)	–	0.118
*P*_*0*_ (mm h^−1^)	*a*_3_ = 1.38 (0.00817)	*b*_3_ = 0.682 (0.00887)	–	0.446
*R*_*e*_ (km)	*a*_4_ = 4.49 (0.00325)	*b*_4_ = 0.271 (0.00475)	*c*_4_ = 0.171 (0.00488)	0.380

The pooled $R^2$ represents the proportion of variance explained when results from all cross-validation folds are combined, providing an overall measure of model fit

Several physical insights emerge from the fitting of Eqs. 3, 4, 5, and 6. $V_{max}$ and TCW are significant positive predictors of $P_m$ (Eq. 3), consistent with the understanding that a stronger TC intensity and a moister atmosphere is conducive to higher TC rain rates (Lonfat et al. 2004; Hill and Lackmann 2009; Lavender and Mcbride 2021; Kim et al. 2022). The negative correlation between $R_m$ and and $P_m$ (Eq. 4) implies a contraction of the rain core with increasing peak rainfall, consistent with eyewall contraction during TC intensification (Willoughby et al. 1982; Stern et al. 2015). The radial gradient $P_m/R_m$ is a significant positive predictor of $P_0$ (Eq. 5), implying that steeper inner-core rainfall gradients are associated with higher central rainfall. Lastly, $R_e$ decreases with $P_m$ but increases with $R_{18}$, implying a faster outer decay of rain for more intense storms but a broader rain field for larger systems.

More generally, these relationships define a hierarchical model structure in which $P_m$, controlled by storm intensity and environmental moisture, governs the spatial organisation of the rainfall field. Higher $P_m$ is associated with a more compact rain core (smaller $R_m$), a steeper core radial gradient (through $P_0$), and a faster outer decay (smaller $R_e$), while the outer rain extent is additionally modulated by storm size ($R_{18}$).

### Post-landfall rain model

Apart from a pre-landfall rain model, a novel post-landfall parametric rain model was also developed and implemented in IRIS. After making landfall, a TC is subjected to substantially greater surface friction, leading to storm decay. This stage is typically characterised by eyewall collapse and the filling of the eye (Lau et al. 2024; Wu et al. 2003). Consequently, the R-CLIPER formulation may not be a realistic depiction of the typical radial structure of the TC rain field after landfall. Observations show that, on average, $R_m$ contracts rapidly after landfall, with the maximum rain rate approaching the TC centre after ~3 h from landfall (Supplementary Fig. S3). Afterwards, rain rates decay approximately exponentially with radius, and the exponential decay model provides an excellent fit to the average radial profile, achieving $R^2 \ge 0.97$ up to 12 h post-landfall (Supplementary Fig. S3). Accordingly, the post-landfall symmetric rain field is modelled as:

7 $$\begin{aligned} P(r, t) = P_c(t)\exp (-\gamma r) \end{aligned}$$

where $P_c$ is the maximum rain rate at the TC centre and $\gamma$ is the radial decay constant. Note that $P_c$ is a function of time after landfall (*t*) that allows for the decay of the rain rates on the land, but $\gamma$ is assumed to be constant throughout the landfall episode. On average, $\gamma$ decreases gradually during the overland period, but the change is modest (< 18% in ~12h; Supplementary Fig. S3). Given the limited number of overland time steps per episode (average ~6, minimum 3), fitting a time-varying $\gamma (t)$ would be highly sensitive to noise and prone to overfitting. From a practical perspective, treating $\gamma$ as constant provides a more robust representation of the radial decay while avoiding over-interpretation of the limited temporal data.

To obtain $P_c$ at the first land point ($P_{c,0}$), a scaling of $P_m$ is employed:

8 $$\begin{aligned} P_{c,0} = P_{m}^\alpha \end{aligned}$$

where $\alpha$ is the scaling parameter for $P_m$. The exponential form provides a simple representation of the non-linear adjustment that occurs after landfall, which slightly outperforms both a linear scaling and a power-law with constant exponent (Supplementary Fig. S4).

The parameter $\gamma$ is not predicted directly but is instead chosen such that integrating Eq. 7 for $t=0$ over the area of the TC yields the storm total volume rate at the first land point ($Vol_{total, 0}$):

9 $$\begin{aligned} Vol_{total, 0} = \iint _{A} P_{c,0}\exp (-\gamma r) \, dA \end{aligned}$$

where *A* is the circular area with a radius of 500 km. The value of $Vol_{total,0}$ is obtained by scaling the total rainfall volume rate of the TC at the last ocean point ($Vol_{total}$):

10 $$\begin{aligned} Vol_{total,0} = Vol_{total}^\beta \end{aligned}$$

where $\beta$ is the scaling parameter for $Vol_{total}$.

The temporal decay of the storm total volume rate over land is modelled as an exponential decay:

11 $$\begin{aligned} Vol_{total}(t) = Vol_{total, 0}\exp (-\delta t) \end{aligned}$$

where $\delta$ is the decay constant. This formulation was found to provide a reasonably good fit to the observed temporal decay of storm total volume rates over land, yielding a pooled $R^2$ of 0.456 (Supplementary Fig. S5). In contrast, the temporal decay of $P_c$ is not modelled directly. Instead, $P_c$ is determined diagnostically such that integrating Eq. 7 over the TC area at each time step yields the corresponding storm total volume rate:

12 $$\begin{aligned} Vol_{total}(t) = \iint _{A} P_{c}(t)\exp (-\gamma r) \, dA \end{aligned}$$

The four parameters (i.e., $\alpha$, $\beta$, $\gamma$, and $\delta$) of the post-landfall rain model were calculated for all global CAT1+ landfall episodes over the analysis period. Only episodes with at least three land points were included, as two-point fits to the volume rate temporal decay (Eq. 11) are always exact, providing no residual degrees of freedom to assess fit quality and resulting in highly unstable decay estimates. After the filtering, a total of 298 episodes were identified worldwide, corresponding to 278 unique TC cases. An illustration of parameter calculation is given in Supplementary Fig. S6.

The volume-focused formulation is adopted because storm-total rain volume is an integral factor in flood risk assessments (Titley et al. 2021; Chakraborty and Mukhopadhyay 2019; Veldhuis et al. 2010). The model explicitly preserves the temporal evolution of storm-total rainfall over land, yielding close agreement with observed post-landfall lifetime rainfall production ($Vol_{lifetime}$) ($R^2=0.995$; Supplementary Fig. S7). The near-perfect agreement arises from the volume-focused modelling framework itself and does not imply similar skill in reproducing other rainfall metrics (e.g., climatological distributions or individual TC rainfall). The model thus provides a suitable basis for applications such as insurance loss modelling and flood planning. By constraining the integrated volume, the formulation also prevents unphysical parameter combinations of rainfall parameters that could otherwise arise if rain rate and size were modelled independently (e.g. an excessively high central rain rate, $P_c$, combined with a very weak radial decay, $\delta$, producing an unrealistically large rain field with persistently extreme rainfall intensities). This constraint therefore enforces a basic conservation constraint and guards against unrealistic outliers, an especially important property for stochastic ensemble simulations. Finally, since storm-total rainfall is more reliably observed than fine-scale spatial patterns, constraining the rainfall field by total volume reduces the number of free parameters and enables robust estimation of model parameters without requiring a full spatio-temporal covariance model for *P*(*r*, *t*).

Following a similar approach to the pre-landfall rain model (Sect. 2.3), $\alpha$ and $\delta$ are predicted by a linear equation:

13 $$\begin{aligned} \alpha&= a_5 + b_5P_m + \epsilon _5 \qquad&\epsilon _5&\sim \mathcal {W}_\textrm{max}(\cdot ) \end{aligned}$$

14 $$\begin{aligned} \delta&= a_6 + b_6LandFrac_{olt} + c_6GH_{olt} + \epsilon _6 \qquad&\epsilon _6&\sim \mathcal {B}(\cdot ) \end{aligned}$$

where *a*, *b*, and *c* are the regression coefficients, and $\epsilon$ is drawn from a distribution. $\mathcal {W}_\textrm{max}$ and $\mathcal {B}$ represent the Weibull Maximum Extreme Value and beta distributions, respectively. In Eq. 14, the subscript “*olt*” denotes the overland trend of the variable, which is the average rate of change of the variable with time during the overland period of the TC. These relationships are empirical but physically consistent (Table 2): the negative dependence of $\alpha$ on $P_m$ reflects a weaker relative scaling for storms with higher peak rain rate (Supplementary Fig. S4), while the dependence of $\delta$ on $LandFrac_{olt}$ and $GH_{olt}$ represents the competing effects of inland decay (via reduced moisture supply and surface roughness) and orographic enhancement of rainfall (Lin et al. 2001).

On the other hand, $\beta$ was not well-constrained by any of the storm or environmental predictors and is thus sampled randomly from a normal distribution fitted to the observed distribution of $\beta$ during the analysis period:

15 $$\begin{aligned} \beta \sim \mathcal {N}(\cdot ) \end{aligned}$$

The stochastic sampling of $\beta$ preserves the observed variability in $Vol_{total,0}$ relative to $Vol_{total}$, and, together with the exponential form of Eq. 10, enables the model to capture the heteroscedastic nature of the variability (Supplementary Fig. S8).

Eqs. 13 and 14 were fitted globally to CAT1+ landfall episodes using least-squares regression. Predictors were standardised by their observed mean and standard deviation (Supplementary Table S1), and the resulting regression coefficients and $R^2$ values are presented in Table 2. The partial regression coefficients are significant at the 95% confidence level. A 5-fold, leave-one-out cross-validation confirmed the robustness of the coefficients, with standard errors less than 1% of the coefficient magnitudes. All noise distributions for $\alpha$ and $\delta$, as well as the sampling distribution for $\beta$, passed the two-sample K-S test at the 5% significance level (Supplementary Fig. S9). The fitted noise parameters, along with the parameters of the sampling distribution for $\beta$, are provided in Supplementary Table S3.

**Table 2:** Estimated regression coefficients for the parameters of the post-landfall rain model defined in Eqs. 13 and 14. As in Table 1

Parameter	*a*	*b*	*c*	Pooled $R^2$
$\alpha$	*a*_5_ = 0.983 (0.00238)	*b*_5_ = −0.0773 (0.00342)	–	0.103
$\delta (h^{-1})$	*a*_6_ = 0.0249 (0.000463)	*b*_6_ = 0.0151 (0.000494)	c_6_ = −0.0110 (0.000602)	0.111

### The storyline

The addition of the proposed rain model to IRIS enables us to evaluate the impacts of global warming on the rainfall of landfalling TCs. To demonstrate this capability, a case study for U.S. hurricanes is performed. To evaluate the thermodynamic impacts of warming on U.S. hurricane rainfall, we follow the storyline approach described and justified in Sparks and Toumi (2025b) and Sparks and Toumi (2025a) to simulate the pre-industrial, present, and future +2 °C climate scenarios. The present climate, defined as the thermodynamic state of 2010, which lies at the midpoint of the analysis period, is used as the control run to validate the proposed rain model integrated into IRIS. A detailed description of the storyline approach is described in Supplementary Text S1. For each climate scenario, a 10,000-year simulation was performed with IRIS. Parent tracks since 1900 were considered in the simulations. While the proposed rain model is validated on the global scale (Sect. 3.1), the case study focuses on CAT1+ landfalls over the U.S. in the NA basin, with particular attention to the impacts of warming on their rainfall (Sect. 3.2). As an illustration, Supplementary Fig. S10 shows the tracks of stochastic TCs making CAT1+ landfalls over the U.S. in the control run, alongside the tracks of historical CAT1+ landfalls within the analysis period.

## Results

### Model validation

**Fig. 1:**
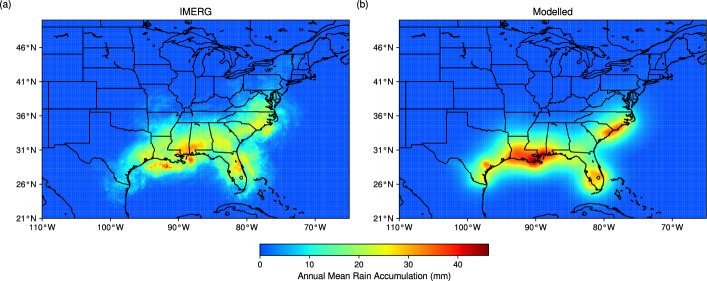
Annual mean accumulated TC rainfall (mm) for hurricane landfalls over the U.S. within the analysis period (1998–2023).** a** shows observations from IMERG and** b** shows the average of 100 realisations of model simulations generated using the proposed parametric rain model. A total of 47 hurricane landfall episodes were identified. Only rain rates within 500 km of the TC centre were considered

To evaluate the performance of the proposed parametric rain model, we followed the approach of Kim et al. (2022) and compared the climatology of annual accumulated rainfall between IMERG observations and the proposed rain model for 47 TC landfall cases over the U.S. (Fig. 1). Owing to the stochastic nature of the model, 100 realisations of the simulated climatology were generated to assess mean model performance and its variability. The results show that the proposed rain model reproduces the observed spatial patterns well, particularly the rainfall maxima along the north-central Gulf Coast (Louisiana to Alabama), Florida, and the Carolina coast. Despite positive biases in magnitude over the Gulf and Carolina maxima, which likely arise from the assumption of a symmetric rainfall field with exponential radial decay that tends to concentrate rainfall near the TC centre compared with the more asymmetric rain fields observed in reality, the mean spatial correlation between observed and simulated rainfall is 0.83 (standard deviation 0.04) for grid points with non-zero accumulated rainfall. The corresponding mean root mean square error (RMSE) is 5.92 mm, with a standard deviation of 1.15 mm . On a per-event basis, the mean spatial correlation is 0.45 (standard deviation 0.18), and the normalised standard deviation, defined as the ratio of the simulated to observed rainfall variability in IMERG, is 1.06 (standard deviation 0.29). These spatial correlations are comparable to, or slightly better than, several existing parametric TC rainfall models (Brackins and Kalyanapu 2020; Marchok et al. 2007). Our model also performs similarly to PHRaMM (Kim et al. 2022) in terms of climatological RMSE and per-event normalised standard deviation, although it shows somewhat weaker skill in pattern matching.

The purpose of IRIS is to assess TC rain risk rather than simulating individual TC events. From a risk assessment standpoint, the return period (RP) and return value (RV) are key metrics for model validation. The RP represents the inverse of the occurrence frequency of events exceeding a given magnitude, known as the RV. Comparisons between IRIS and observed RPs have shown that IRIS generally reproduces the observed last-ocean point $V_{max}$ prior to landfall across global locations (Sparks and Toumi 2024). Specifically for the U.S., both basin-wide LMI $V_{max}$ and landfall $V_{max}$ have been validated in Sparks and Toumi (2025b). In this section, we extend this validation framework to assess the ability of IRIS to reproduce observed TC rainfall risk.

**Fig. 2:**
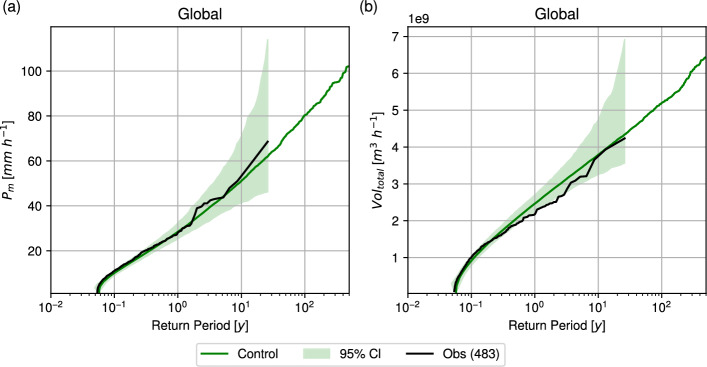
Global validation of the IRIS model for the **a** $P_m$ (mm h^−1^) and **b** $V_{total}$ (m^3^ h^−1^) at the last ocean point before landfall. Shown are return period curves from the 10,000-year IRIS control run (green) and from observations for 1998–2023 (black, with the number of events indicated in parentheses in the legend). The green shading indicates the 95% confidence interval, estimated from bootstrapping 1,000 26-year samples of the IRIS control run to match the observational record length

First, the performance of IRIS was validated at the last ocean point prior to landfall. Figure 2 demonstrates that IRIS can generate events beyond the observed range and shows good agreement with the observed $P_m$ and $Vol_{total}$, with observed RPs falling within the 95% confidence interval derived by bootstrapping 26-year samples from the control simulation. Validation at the national scale (Supplementary Fig. S11) likewise indicates generally good agreement between IRIS and the observations. Nonetheless, regional discrepancies are evident: for instance, observed $Vol_{total}$ values for India are markedly lower than those from IRIS, whereas for Japan they are higher. These differences may reflect regional model bias or sampling uncertainty in the observational record, bearing in mind the limited number of observational records available within the analysis period and the fact that the observations represent only one realisation of the range of plausible historical outcomes. IRIS has also been validated for $V_{max}$, $P_0$, $R_m$, and $R_e$ at the last ocean point before landfall (Supplementary Fig. S12).

**Fig. 3:**
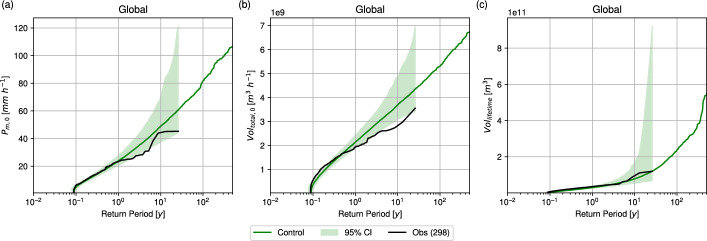
Global validation of the IRIS model for the** a** maximum azimuthal mean rain rate (mm h^−1^) and** b** storm-total volume rate (m^3^ h^−1^) at the first land point after landfall and** c** lifetime volume production over land. Elements are as in Fig. 2

Following the validation of pre-landfall rain risk, the post-landfall rain risk in IRIS was also assessed. Figure 3 compares the RPs of $P_{m,0}$, $Vol_{total,0}$, and $Vol_{lifetime}$ between the IRIS control run and observations. The results indicate reasonably good agreement, with observed $P_{m,0}$ and $Vol_{total,0}$ generally towards the lower end of the simulated return values. As with the pre-landfall validation, post-landfall rain risk was also evaluated at the country level (Supplementary Fig. S13). For the six countries shown, observed $P_{m,0}$ mostly lies within the 95% confidence interval of IRIS, although for the U.S. some return values fall below the $2.5^\textrm{th}$ percentile. For $Vol_{total,0}$, observed return values exceed those of IRIS in some countries (e.g., Japan and the Philippines) and are lower in others (e.g., India), but generally remain within the 95% confidence interval. Observed $Vol_{lifetime}$ return values also compare favourably with IRIS, with most curves lying within the 95% confidence interval. Overall, these results demonstrate that, through implementation of the proposed parametric rain model (Sects. 2.3–2.4), IRIS reproduces the observed rain risk associated with TC landfall reasonably well and is therefore suitable for assessing TC rainfall hazard and risk. It is noteworthy that, although the parameters and noise distributions of the rainfall model were fitted using observations, IRIS can also simulate extreme events with very long return periods (> 100 years) that are not present in the historical record. This arises from the stochastic nature of the model framework, which allows extreme values of different parameters to combine in ways that may not have been observed historically.

### Case study for the U.S.

#### Return period curves

This section presents a case study of the historical and future changes of U.S. hurricane rainfall, illustrating the capability of IRIS with the incorporated parametric rain model.

**Fig. 4:**
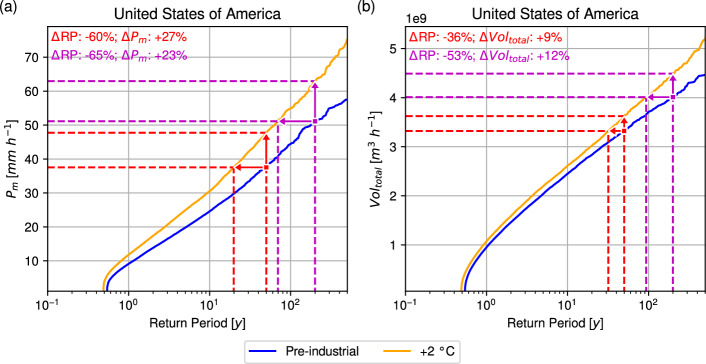
Return curves for IRIS simulations of pre-industrial (blue) and +2 °C (orange) simulations for **a** $P_m$ (mm h^−1^) and **b** $V_{total}$ (m^3^ h^−1^) of U.S. landfalling hurricanes. Red (Magenta) lines and arrows show the impact of +2 °C on a 50- (200-) year event

For the last ocean point prior to landfall, the impacts of a +2 °C scenario on $P_m$ and $Vol_{total}$ are shown in Fig. 4. The figure shows that under the +2 °C scenario, both RP curves for $P_m$ and $Vol_{total}$ shift upward, with progressively larger changes at longer RPs. For $P_m$, the pre-industrial 50-year event becomes a 19.8-year event, representing a 60.3% reduction in RP. Another perspective is that the $P_m$ of the 50-year event increases by 10.2 mm h^−1^, a 27.1% increase. For the pre-industrial 200-year event, the RP shortens to 69.5 y, a 65.2% decrease, while the $P_m$ increases by 11.9 mm h^−1^, or 23%. Overall, there is a 20.1% increase in $P_m$ on average. For $Vol_{total}$, a 50-year event in the pre-industrial climate becomes a 31.9-year event (36.2% decrease). In equivalent terms, the $Vol_{total}$ of the 50-year event rises by 3 x 10^9^ m^3^ h^−1^, or 9.1%. The effect on the 200-year event is greater, with the RP halving to 93.6 y (53% decrease), while $Vol_{total}$ increases by 4.8 x 10^9^ m^3^ h^−1^, or 11.9%. On average a 3.9% increase in $Vol_{total}$ is observed. Focusing on the x-intercepts of the curves in either panel, landfall frequency increases in the +2 °C scenario. Although the mean basin frequency is held fixed (Sparks and Toumi 2025b), landfall frequency rises from 1.8 y to 2.0 y, an increase of 11.0%. This occurs because the LMI is higher in the warmer climate (i.e., higher initial TC intensities), allowing more TCs to survive the decay process and reach landfall with CAT1+ intensity.

**Fig. 5:**
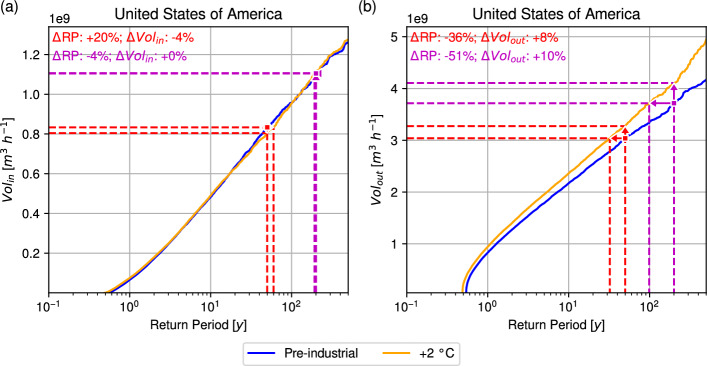
Return curves for IRIS simulations of pre-industrial (blue) and +2 °C (orange) simulations for the** a** inner rain volume rate (m^3^ h^−1^) and** b** outer rain volume rate (m^3^ h^−1^) of U.S. landfalling hurricanes at the last ocean point before landfall. Red (Magenta) lines and arrows show the impact of +2 °C on a 50- (200-) year event. The inner rain volume rate is computed within the $R_m$ of the TC, while the outer rain volume rate is computed between $R_m$ and 500 km

An important finding is that the rain volume rate response to warming is not spatially uniform but shows a marked asymmetry between the inner core and the outer region of the hurricane. The $R_m$ is used to divide the rain field into an inner region ($r \le R_m$) and an outer region ($R_m< r < 500\ \textrm{km}$). Figure 5 presents the RP curves for the inner rain volume rate ($Vol_{in}$) and the outer rain volume rate ($Vol_{out}$) at the last ocean point before landfall. The results indicate minimal change in $Vol_{in}$ between the pre-industrial and +2 °C scenarios: for a 50-year event, $Vol_{in}$ decreases slightly by 3.5%, while for a 200-year event it remains nearly unchanged (+0.2%). On average, $Vol_{in}$ declines by 5.0% under warming. By contrast, $Vol_{out}$ increases substantially, with gains of 8.0% for the 50-year event and 10.0% for the 200-year event, corresponding to an overall average increase of 5.1%. These results suggest that the increase in $V_{total}$ under warming is primarily driven by growth in $Vol_{out}$, with changes in $Vol_{in}$ partly offsetting this increase. Further analysis indicates that the reduction in $Vol_{in}$, despite higher $P_m$ (Fig. 4) and $P_0$ (Supplementary Fig. S14a), is attributable to a smaller $R_m$ under warming (Supplementary Fig. S14b).

**Fig. 6:**
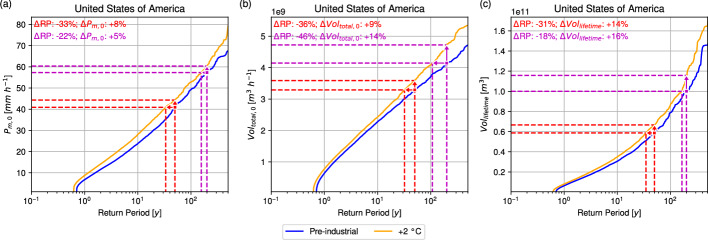
Return curves for IRIS simulations of pre-industrial (blue) and +2 °C (orange) simulations for **a** $P_{m,0}$ (mm h^−1^),**b** $Vol_{total,0}$ (m^3^ h^−1^), and** c** $Vol_{lifetime}$ (m^3^) of U.S. landfalling hurricanes. Red (Magenta) lines and arrows show the impact of +2 °C on a 50- (200-) year event

Next, the impact of the +2 °C scenario on TC rainfall after landfall is assessed. Figure 6 presents the RP curves for $P_{m,0}$, $Vol_{total,0}$, and $Vol_{lifetime}$ for hurricanes following landfall over the U.S.. At the first land point, both $P_{m,0}$ and $Vol_{total,0}$ increase across RPs under warming. For $P_{m,0}$, the pre-industrial 50-year event shortens to 33.5 years in the +2 °C scenario, a reduction of 33.1%. Equivalently, the $P_{m,0}$ of a 50-year event rises by 3.4 mm h^−1^ (8.3%). For the 200-year event, the RP decreases to 156.2 years (21.9% shorter), with $P_{m,0}$ increasing by 3.1 mm h^−1^ (5.4%). On average, $P_{m,0}$ increases by 1.5 mm h^−1^ (12.7%) under warming. For $Vol_{total,0}$, the pre-industrial 50-year event becomes a 32.2-year event, a 35.7% reduction in RP, corresponding to an increase of 2.98 × 10^9^ m^3^ h^−1^ (9.1%). At the 200-year RP, the return period halves to 107.3 years (46.3% shorter), with $Vol_{total,0}$ increasing by 5.8 × 10^9^ m^3^ h^−1^ (13.9%). On average, $Vol_{total,0}$ increases by 3.8 × 10^7^ m^3^ h^−1^ (3.4%) in the warmer climate. $Vol_{lifetime}$ also shows pronounced increases across RPs under warming. The pre-industrial 50-year event is reduced to a 34.6-year RP, a decrease of 30.8%, corresponding to an increase of 8.0 × 10^10^ m^3^ (13.6%). For the 200-year event, the RP falls to 164.9 years (17.6% shorter), while $Vol_{lifetime}$ increases by 1.6 × 10^11^ m^3^ (15.7%). On average, $Vol_{lifetime}$ increases by 2.0 × 10^9^ m^3^ (14.6%) under the +2 °C scenario.

#### Maps of variables at specified return periods

To explore the spatial variation of the impact of warming on U.S. hurricane rainfall, a series of return value maps for rain rate and accumulated rain volume are drawn for the East and Gulf Coast of the U.S..

**Fig. 7:**
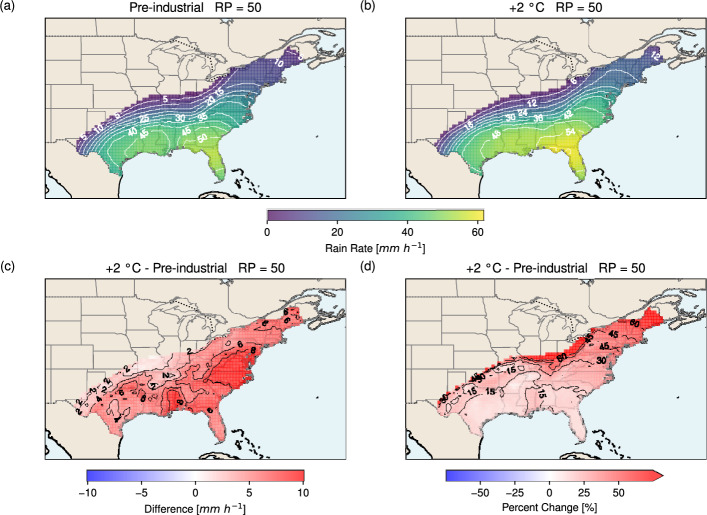
Hurricane rain rate (mm h^−1^) maps at the 50-year return period over the U.S. for** a** the pre-industrial and** b** the +2 °C scenario. Panel** c** shows the difference between the two scenarios (+2 °C minus pre-industrial), and panel** d** the corresponding percentage change. Return values are computed on a regular latitude–longitude grid at 0.5° resolution, using rain rates within 500 km of the TC centre. Only CAT1+ landfalls over the U.S. are considered. TC instances at the last ocean point before landfall and over land points above TS strength (> 17.5 m s^−1^ ) are included. For the last ocean point (land points), Eqs. 1 and 2 (Eq. 7) are (is) applied to construct the TC rain field

Figure 7 shows the rain rate map at the 50-year RP. In both the pre-industrial and +2 °C scenarios (Fig. 7a, b), the highest rain rates occur in Florida (> 50 mm h^−1^ for pre-industrial; > 54 mm h^−1^ for +2 °C). Rain rates decrease northward, with a steeper decline inland and a more gradual decay along the East Coast. The difference map (Fig. 7c) shows that rain rates increase everywhere under warming, with the largest absolute increases (> 8 mm h^−1^) along the Alabama–Mississippi Gulf Coast and the North Carolina–Virginia East Coast. On average, rain rates in the +2 °C scenario are 5.4 mm h^−1^ higher than in the pre-industrial climate. In relative terms, the largest percentage increases (> 60%) occur inland and at the north-eastern tip of the East Coast (Fig. 7d), primarily because rain rates in these regions are low in the pre-industrial scenario, so even modest absolute increases in the +2 °C scenario produce large fractional changes. The average relative increase is 38.0%.

**Fig. 8:**
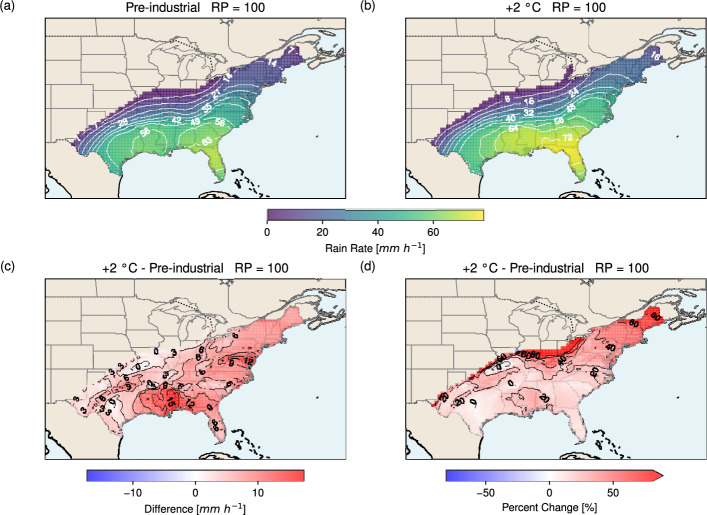
As in Fig. 7 but for a return period of 100 years

The rain rate map for the 100-year RP is shown in Fig. 8. The spatial pattern for the pre-industrial scenario (Fig. 8a) is broadly similar to that of the 50-year RP, with the highest rain rates located in Florida (> 63 mm h^−1^) and decreasing northward, with a secondary maximum over Louisiana (> 56 mm h^−1^). In the +2 °C scenario (Fig. 8b), a single maximum is found over northern Florida (> 72 mm h^−1^). The warmer climate also shows deeper inland penetration of TC rainfall, with non-zero values extending as far north as Michigan. The difference map (Fig. 8c) highlights widespread increases, with the most pronounced rise (> 15 mm h^−1^) over Mississippi and Alabama. On average, rain rates increase by 6.2 mm h^−1^ under warming. In relative terms (Fig. 8d), the largest percentage increases (> 80%) occur inland and at the north-eastern tip of the East Coast, similar to that of the 50-year RP. The average percentage increase is 29.3%.

**Fig. 9:**
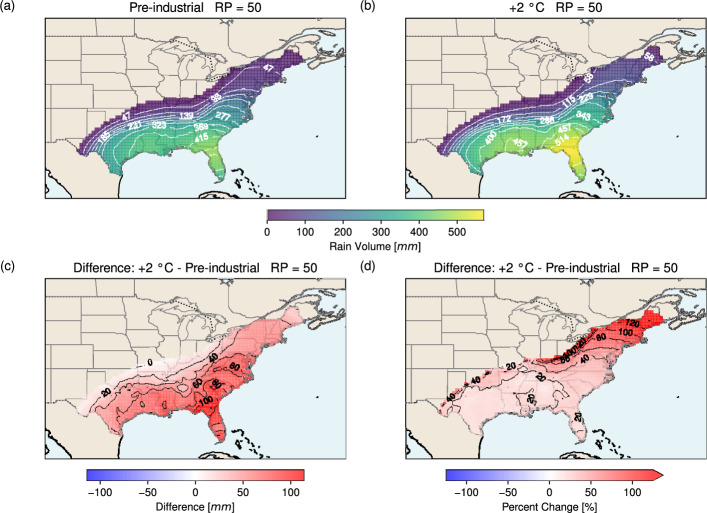
24-hour rain volume (mm) due to a hurricane at the 50-year return period over the U.S. for** a** the pre-industrial scenario and** b** the +2 °C scenario. Panel** c** shows the difference between the two scenarios (+2 °C minus pre-industrial), and panel** d** the corresponding percentage change

In addition to rain intensity, the total amount of rainfall (i.e., the accumulated rain volume) is a key factor controlling flood risk. Figure 9 presents the 24-hour rain volume map at the 50-year RP over the U.S. In both the pre-industrial and +2 °C scenarios (Fig. 9a, b), the maximum volumes occur in Florida (> 415 mm for pre-industrial; > 514 mm for +2 °C). As with rain rates (Figs. 7 and 8), rain volume decreases northward, with a sharper decline inland and a more gradual reduction along the East Coast. The difference map (Fig. 9c) shows greater volumes everywhere under warming, with the largest increase (> 100 mm) along the Florida–Georgia border. Increases are generally stronger in coastal areas and weaker inland. On average, rain volume increases by 49.2 mm. In percentage terms (Fig. 9d), the largest relative increases (> 120%) occur inland and at the north-eastern tip of the East Coast, while lower increases are found further south, mirroring the pattern seen for rain rate (Fig. 7). On average, the 24-hour rain accumulation increases by 41.8% under warming.

**Fig. 10:**
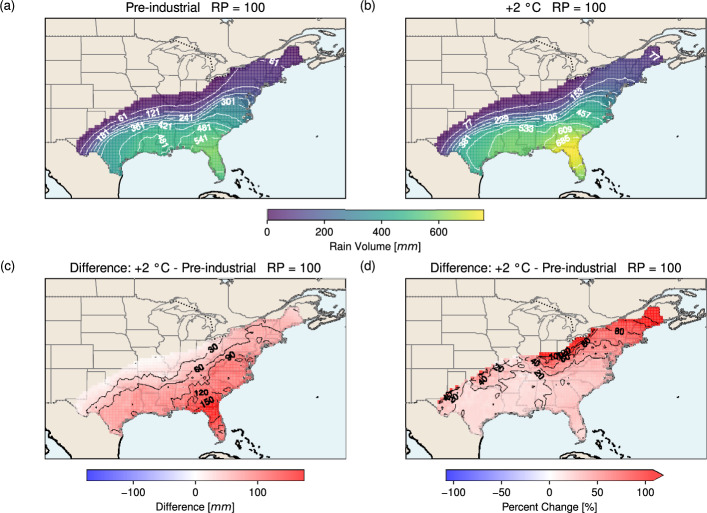
As in Fig. 9 but for a return period of 100 years

At the 100-year RP, the spatial patterns of rain volume in the pre-industrial and +2 °C scenarios (Fig. 10a, b) closely resemble those of the 50-year RP (Fig. 9a, b), though with substantially larger magnitudes. The maximum volume exceeds 541 mm in the pre-industrial climate and 685 mm in the +2 °C scenario, both located in Florida. Under warming, large increases are seen across the south-eastern to eastern U.S., from Florida to Virginia, with the strongest rise (> 100 mm) in this region. On average, the 24-hour volume increases by 63.7 mm. In relative terms, the pattern mirrors that of the 50-year RP (Fig. 9d), with the greatest increases (> 120%) inland and at the north-eastern tip of the East Coast. On average, the 24-hour rain accumulation in the +2 °C scenario is 41.2% greater than in the pre-industrial climate.

## Discussion

With global warming, the thermodynamic expectation for the rate of rainfall increase is about 7% °C^−1^, as derived from the C–C relation. Thus, a surface temperature rise of 2 °C would imply an expected 14% increase in rainfall. However, numerous studies have reported higher rates of increase (Kim et al. 2022; Wright et al. 2015; Knutson et al. 2015; Liu et al. 2019; Yang and Toumi 2025; Chen et al. 2025; Knutson et al. 2022, 2013), which may be linked to the projected intensification of TCs under warming (Liu et al. 2019). In the present study, we also find evidence of “super C–C” scaling of approximately 10% °C^−1^ (Sect. 3.2.1) for the $P_m$ of hurricanes approaching landfall in the U.S.. Using the set-up of Knutson et al. (2013), Wright et al. (2015) performed dynamic downscaling experiments using the 18-km Zetac RCM for 27 seasons of August–October Atlantic-basin hurricane activity for a control period of 1980–2006 and three climate changes scenarios. They reported an increase of around 16% in maximum azimuthal mean rainfall rate over the ocean under a warming of ~1.7 °C under the CMIP3 SRES A1B scenario (their Fig. 7, right panels), which compares favourably to our result. In addition, we find that the post-landfall maximum azimuthal mean rainfall rate ($P_{m,0}$) of U.S. landfalling hurricanes increases by about 6.3% °C^−1^ (Sect. 3.2.1), which is lower than the C–C scaling. This represents a sub C–C response and agrees with Wright et al. (2015) (their Fig. 7, left panels), who also reported sub-Clausius–Clapeyron scaling in both the CMIP3 SRES A1B (~5.9% °C) and late CMIP5 RCP4.5 (~3.8% °C^−1^) scenarios for the maximum azimuthal mean rainfall rate over land.

In terms of near-storm average rain rate, a recent work by Knutson et al. (2022) adopted the downscaling approach of Knutson et al. (2013) and Wright et al. (2015) but performed a further dynamical downscaling of individual storms using the 9-km Geophysical Fluid Dynamics Laboratory hurricane model. For TCs making U.S. landfalls, they found a multi-model mean increase of 18% for rain rates averaged within 100 km of the TC centre under warming scenarios of ~1.7 °C. In the current study, we found a percentage increase of 15.3% in the +2 °C scenario, which is a smaller increase compared to Knutson et al. (2022) but still exceeds the C–C expectation. Using an averaging radius of 150 km, Wright et al. (2015) found mean increases of 12% over the ocean and 6% over land. Our analysis yields corresponding increases of 13.1% and 5.8%, respectively, in excellent agreement with their results. The increase over the ocean also aligns closely with the median change (+14%) from 16 global GCM/RCM projections across eight studies reviewed by Knutson et al. (2020). For a larger averaging radius of 500 km, we find mean increases of 5.8% over the ocean and 5.6% over land, smaller than the 10% and 12% increases reported by Wright et al. (2015) but within the global projection range of +3.1–+22% summarised by Knutson et al. (2020).

With respect to the spatial variability in the response of TC rain to warming, a notable feature in Figs. 7, 8 is the local maximum percentage increase in rain rate over Mississippi–Alabama. Wright et al. (2015) found a local maximum increase of the average August–October rainfall in the same region for all three warming scenarios (their Fig. 4), despite a slight decline in track density (their Fig. 2). A more direct comparison is provided in Supplementary Fig. S15, which compares the the average August–October rainfall in the +2 °C scenario with the pre-industrial scenario in IRIS. Comparing Supplementary Fig. S15d with their Fig. 4 (right panels) reveals qualitative similarities: (a) a local maximum increase over Mississippi–Alabama, (b) heavier rainfall over North Carolina, Tennessee, and Virginia under warming, and (c) weaker increases (decreases for Wright et al. (2015)) over the Florida–Georgia region. However, the large increase seen in northeastern U.S. is absent in Wright et al. (2015), likely due to the artificial dissipation of TCs as they approach the northern boundary of their model domain (45 °N).

Overall, the integration of the proposed parametric rain model into IRIS demonstrates that the framework can generate future TC rain projections consistent with full-physics GCMs and RCMs in the U.S. Unlike these computationally demanding approaches, IRIS can provide global projections efficiently while maintaining good agreement with observed rain risk and showing no clear systematic bias. As direct estimation of TC rain risk from observations is limited by the infrequency of TC events and uneven observational coverage, IRIS overcomes these constraints by generating hundreds of thousands of synthetic TC tracks. This enables the estimation of TC rain risk at a higher spatial and temporal resolution, including the estimation of long return periods of extreme TC rainfall events and provision of risk assessments for inland locations where observations are lacking. Although this study focused on the U.S. as a case study, the IRIS framework can be readily applied to estimate TC rain risk globally, but further evaluation is needed to assess its performance outside the U.S.

TC rainfall characteristics can vary substantially between ocean basins, and some of the regional biases identified in this study likely reflect these differences (Supplementary Figs. S11 and S13). In principle, basin-specific model development or regional bias correction could improve performance. However, the relatively limited number of TC landfall samples available in the observational record makes such approaches difficult. Partitioning the dataset by basin would substantially reduce the number of samples available for parameter estimation, particularly in basins with relatively few TCs (e.g., the North Indian basin), which may lead to poorly constrained parameters and increased risk of overfitting. For this reason, the rainfall model was fitted globally to maximise the available sample size and then evaluated across regions. The biases identified over the U.S., namely the negative bias of RPs for several parameters (Supplementary Figs. S11 and S13), warrant caution when interpreting the absolute magnitude of simulated extremes. For climate projections (Sect. 3.2), however, the primary quantity of interest is the relative change in TC rainfall risk between scenarios. Because the same modelling framework is applied consistently across climate scenarios, systematic biases are expected to affect both simulations similarly and are therefore unlikely to substantially influence the estimated fractional changes in risk. Regional bias correction may nevertheless be explored in future work as longer observational records or larger datasets become available.

Although TC rain rates are projected to increase significantly with warming, the storm-total rain volume rate shows a weaker sensitivity. We find that the 500-km volume rate increases by only ~2% °C^−1^ at the last ocean point and ~1.7% °C^−1^ at the first land point (Sect. 3.2.1), both well below the Clausius–Clapeyron expectation. This weaker response likely reflects the shrinkage of $R_m$ (~5.3% °C^−1^) and $R_e$ (~3.4% °C^−1^) in the +2 °C scenario (Supplementary Fig. S14b, c). Although $P_m$ increases, the more compact rain field offsets gains in storm-total volume. Similar mechanisms were identified by Yang and Toumi (2025), who found super-C–C scaling of $P_m$ but a weaker scaling for rain volume due to $R_m$ contraction using a convection-permitting model, and by Chen et al. (2025), who also identified super-C–C scaling of $P_m$ and $P_0$ but shrinkage of $R_m$ and $R_e$ in MRI-HighRes CMIP6 SSP5−8.5 simulations using a GCM, despite weaker sensitivities of −1.03% °C and −1.79% °C. Taken together, these findings show that our statistical rain model is in good agreement with both convection-permitting models and GCMs in reproducing a robust feature of warming: the contraction of TC rain fields, which constrains increases in storm-total rainfall despite stronger peak rates.

Despite its advantages, the IRIS approach has several limitations. The current rainfall models simulate only the axisymmetric component of the TC rain field. Mesoscale features such as spiral rainbands and individual convective cells are not represented, making the model unsuitable for reproducing rainfall from individual events. For risk assessment purposes, however, these features are expected to average out across ensembles of storms (Zhu et al. 2013). It is also well established that TC rainfall often exhibits pronounced spatial asymmetries, driven by factors such as environmental vertical wind shear (Chen et al. 2006), storm translation (Lonfat et al. 2004), and topography (Huang et al. 2020). These asymmetries are likewise not captured by IRIS, further limiting its application to single-event rainfall estimation. Nevertheless, Brackins and Kalyanapu (2020) evaluated four parametric TC rainfall models against observed precipitation from 67 Atlantic storms affecting the U.S. and found comparable performance between models that include asymmetry and those that do not. Specifically, the R-CLIPER model, on which our pre-landfall formulation is based, performed nearly identically to the PHRaM model across indices of pattern matching, mean, median, and volumetric rainfall, as well as extremes (see Table 8 of Brackins and Kalyanapu (2020)), despite PHRaM incorporating additional asymmetry components. On a per-event basis, the rainfall pattern correlation of our model (0.45) is comparable to that of the best-performing model (0.467) evaluated by Brackins and Kalyanapu (2020) (their Fig. 5). PHRaMM (Kim et al. 2022), which accounts for shear- and topography-induced asymmetry, performs better in simulating both climatological and event-based rainfall for WNP landfalling TCs. However, that model was optimised specifically for the WNP, whereas IRIS was fitted globally. Furthermore, Kim et al. (2022) considered over-ocean TCs with centres within 300 km of the coastline as part of the landfall period, whereas our analysis includes only the last ocean point. Consequently, our dataset contains proportionally more overland grid points, which are more difficult to simulate due to abrupt structural changes as TCs weaken following landfall.

Another limitation of the IRIS approach is that it does not account for extratropical transition (ET). ET is known to induce substantial changes in the rainfall structure of TCs (Jones et al. 2003; Chen 2011; Atallah and Bosart 2003). It should therefore be noted that some synthetic tracks simulated by IRIS at higher latitudes could undergo ET if they occurred in reality. For these cases, IRIS may not reproduce the realistic evolution of rainfall. Over the continental U.S., ET has a limited influence on storms with a CAT1+ LMI below 35 °N, where extratropical systems account for no more than 10% of storms (Supplementary Fig. S16a). Beyond 35 °N, the proportion of extratropical systems increases sharply, exceeding 50% above 40 °N (Supplementary Fig. S16a). Consequently, caution should be exercised when interpreting results for tracks at higher latitudes (> 35  °N) where ET is more likely. Work is currently underway to incorporate the capability to simulate ET within IRIS.

In our U.S. case study, we adopted the storyline approach of Sparks and Toumi (2025b) to generate future projections. This approach is motivated by the higher confidence in the thermodynamic increases of PI (Pérez-Alarcón et al. 2023) and TCW (Borger et al. 2022), in contrast to the deep uncertainty surrounding dynamical changes projected by climate models (Knutson et al. 2020; Sobel et al. 2023; Shepherd et al. 2018). As discussed in detail in Sparks and Toumi (2025b), while the storyline framework does not provide a complete representation of future variability, it serves as a valuable tool for isolating the thermodynamic contribution—the component in which confidence is greatest. Key dynamical factors (e.g. vertical wind shear and steering flow) and their potential impacts on TC frequency, decay, and track were not explicitly modelled. However, sensitivity experiments in Sparks and Toumi (2025b) show that their changes up to the magnitude of their decadal variability are insufficient to counteract the increased risk of intense hurricanes associated with enhanced PI. Since $P_m$ is directly governed by $V_{max}$ in the proposed model and, in turn, influences the other rainfall parameters, we therefore expect that dynamical changes would be insufficient to offset the projected increase in rain risk.

## Conclusion

In this study, a parametric rain model has been developed to represent the rain field of landfalling TCs. By integrating this model into IRIS, a statistical-thermodynamic TC hazard model, it has been shown that the model achieves comparable or slightly superior skill to existing parametric approaches in reproducing observed rainfall climatology and event-scale rainfall. The model also captures the observed global return period curves of maximum azimuthal mean rain rate and storm-total volume rate both before and after landfall, as well as the lifetime rain volume over land. Incorporating the rain model into IRIS enables the projection of future TC rainfall risk on a global scale. This capability has been demonstrated through a case study for the U.S. Under thermodynamic changes in PI and TCW associated with a +2 °C warming, landfalling hurricanes exhibit higher maximum azimuthal mean rain rates, increased storm-total rain volumes, and greater lifetime rainfall over land. The contraction of the rain field with warming is also captured, leading to a smaller increase in storm-total volume than in peak rain rate. The spatial variability of projected rainfall risk was assessed using rain rate and volume maps at specified return periods. A +2 °C warming is found to increase both rain rates and 24-hour rainfall accumulations across the eastern and southern U.S., with the largest absolute increases in the southeast. However, the greatest relative increases occur inland and in the northeast, indicating enhanced inland and poleward penetration of hurricane rainfall under warming, which may intensify rainfall hazards in regions not typically exposed to such extremes.

Future work could examine whether incorporating a parametric representation of the asymmetric component of the TC rain field would lead to a substantial improvement in model performance. In addition, exploring regional biases in the model could provide further insight into the controlling factors of TC rainfall at landfall.

## Supplementary Information

Below is the link to the electronic supplementary material.

Supplementary file 1 (pdf 8126 KB)

## References

[CR1] Atallah EH, Bosart LF (2003) The extratropical transition and precipitation distribution of Hurricane Floyd (1999). Mon Weather Rev 131(6):1063–1081

[CR2] Bister M, Emanuel KA (2002) Low frequency variability of tropical cyclone potential intensity 1. interannual to interdecadal variability. J Geophys Res: Atmospheres 107(D24):ACL–26. Publisher: Wiley Online Library

[CR3] Bloemendaal N, Haigh ID, de Moel H et al (2020) Generation of a global synthetic tropical cyclone hazard dataset using STORM. Scientific Data 7(1):40. 10.1038/s41597-020-0381-2 32029746 10.1038/s41597-020-0381-2 PMC7005259

[CR4] Borger C, Beirle S, Wagner T (2022) Analysis of global trends of total column water vapour from multiple years of OMI observations. Atmos Chem Phys 22(16):10603–10621. 10.5194/acp-22-10603-2022

[CR5] Brackins JT, Kalyanapu AJ (2020) Evaluation of parametric precipitation models in reproducing tropical cyclone rainfall patterns. J Hydrol 580:124255. https://www.sciencedirect.com/science/article/pii/S0022169419309904

[CR6] Chakraborty S, Mukhopadhyay S (2019) Assessing flood risk using analytical hierarchy process (AHP) and geographical information system (GIS): application in Coochbehar district of West Bengal. India Natural hazards 99(1):247–274

[CR7] Chen G (2011) A comparison of precipitation distribution of two landfalling tropical cyclones during the extratropical transition. Adv Atmos Sci 28(6):1390–1404

[CR8] Chen SS, Knaff JA, Marks FD Jr (2006) Effects of vertical wind shear and storm motion on tropical cyclone rainfall asymmetries deduced from TRMM. Mon Weather Rev 134(11):3190–3208

[CR9] Chen J, Toumi R, Zhang L, et al (2025) Radial rainfall pattern changes of intense over-ocean tropical cyclones under global warming: insights from an MRI HighRes CMIP6 simulation. Geophys Res Lett 52(9):e2025GL116146. 10.1029/2025GL116146, https://onlinelibrary.wiley.com/doi/abs/10.1029/2025GL116146, _eprint: https://agupubs.onlinelibrary.wiley.com/doi/pdf/10.1029/2025GL116146

[CR10] Clarke B, Li S, Toumi R, et al (2025) The influence of anthropogenic climate change on Super Typhoon Odette (Typhoon Rai) and its impacts in the Philippines. EGUsphere pp 1–56. https://doi.org/10.5194/egusphere-2025-665, https://egusphere.copernicus.org/preprints/2025/egusphere-2025-665/, publisher: Copernicus GmbH

[CR11] Collalti D, Strobl E (2022) Economic damages due to extreme precipitation during tropical storms: evidence from Jamaica. Nat Hazards 110(3):2059–2086

[CR12] Emanuel K (2006) Climate and tropical cyclone activity: a new model downscaling approach. J Clim. 10.1175/JCLI3908.1

[CR13] Emanuel K (2017) Assessing the present and future probability of Hurricane Harvey’s rainfall. Proc Natl Acad Sci 114(48):12681–12684. 10.1073/pnas.1716222114 29133388 10.1073/pnas.1716222114 PMC5715789

[CR14] Emanuel KA (1986) An air-sea interaction theory for tropical cyclones. Part I: steady-state maintenance. J Atmos Sci 43(6):585–605

[CR15] Gilford DM (2021) pyPI (v1. 3): Tropical cyclone potential intensity calculations in Python. Geoscientific Model Development 14(5):2351–2369. Publisher: Copernicus Publications Göttingen, Germany

[CR16] Gori A, Lin N, Xi D et al (2022) Tropical cyclone climatology change greatly exacerbates US extreme rainfall–surge hazard. Nat Clim Chang 12(2):171–178. https://www.nature.com/articles/s41558-021-01272-7, publisher: Nature Publishing Group

[CR17] Guzman O, Jiang H (2023) Climatology of tropical cyclone rainfall magnitude at different landfalling stages: an emphasis on after-landfall rain. J Appl Meteorol Climatol 62(7):801–815. 10.1175/JAMC-D-22-0055.1

[CR18] Guzman O, Jiang H (2021) Global increase in tropical cyclone rain rate. Nat Commun 12(1):5344 Publisher: Nature Publishing Group UK London 10.1038/s41467-021-25685-2 PMC8429722 34504083

[CR19] Hersbach H, Bell B, Berrisford P et al (2020) The ERA5 global reanalysis. Q J R Meteorol Soc 146(730):1999–2049

[CR20] Hersbach H, Bell B, Berrisford P, et al (2023) ERA5 hourly data on single levels from 1940 to present. 10.24381/cds.adbb2d47, type: Dataset

[CR21] Hill KA, Lackmann GM (2009) Influence of environmental humidity on tropical cyclone size. Mon Weather Rev 137(10):3294–3315

[CR22] Hill KA, Lackmann GM (2011) The impact of future climate change on TC intensity and structure: a downscaling approach. J Clim. 10.1175/2011JCLI3761.1

[CR23] Huang CY, Chou CW, Chen SH et al (2020) Topographic rainfall of tropical cyclones past a mountain range as categorized by idealized simulations. Weather Forecast 35(1):25–49

[CR24] Huffman GJ, Bolvin DT, Braithwaite D, et al (2023) Nasa global precipitation measurement (GPM) integrated multi-satellite retrievals for GPM (IMERG) version 07. Algorithm Theoretical Basis Document (ATBD) Version 47

[CR25] James MK, Mason LB (2005) Synthetic tropical cyclone database. J Waterw Port Coast Ocean Eng 131(4):181–192. 10.1061/(ASCE)0733-950X(2005)131:4(181)https://ascelibrary.org/doi/10.1061/%28ASCE%290733-950X%282005%29131%3A4%28181%29 publisher: American Society of Civil Engineers

[CR26] Jones SC, Harr PA, Abraham J et al (2003) The extratropical transition of tropical cyclones: forecast challenges, current understanding, and future directions. Weather Forecast 18(6):1052–1092

[CR27] Kamahori H (2012) Mean Features of tropical cyclone precipitation from TRMM/3B42. Sola 8:17–20. 10.2151/sola.2012-005

[CR28] Kim D, Park DSR, Nam CC, et al (2022) The parametric hurricane rainfall model with moisture and its application to climate change projections. npj Climate and Atmospheric Science 5(1):86. Publisher: Nature Publishing Group UK London

[CR29] Kim HS, Vecchi GA, Knutson TR, et al (2014) Tropical cyclone simulation and response to CO2 doubling in the GFDL CM2.5 high-resolution coupled climate model. J Climate https://doi.org/10.1175/JCLI-D-13-00475.1, https://journals.ametsoc.org/view/journals/clim/27/21/jcli-d-13-00475.1.xml

[CR30] Knapp KR (2024) International best track archive for climate stewardship (IBTrACS) technical documentation. Report, NOAA National Centers for Environmental Information, https://www.ncei.noaa.gov/sites/g/files/anmtlf171/files/2024-07/IBTrACS_version4r01_Technical_Details.pdf

[CR31] Knutson T, Camargo SJ, Chan JCL et al (2020) Tropical cyclones and climate change assessment: part II: projected response to anthropogenic warming. Bulletin Am Meteorol Socie. 10.1175/BAMS-D-18-0194.1.

[CR32] Knutson TR, Sirutis JJ, Vecchi GA et al (2013) Dynamical downscaling projections of twenty-first-century Atlantic Hurricane activity: CMIP3 and CMIP5 model-based scenarios. J Clima. https://doi.org/10.1175/JCLI-D-12-00539.1. https://journals.ametsoc.org/view/journals/clim/26/17/jcli-d-12-00539.1.xml

[CR33] Knutson TR, Sirutis JJ, Zhao M, et al (2015) global projections of intense tropical cyclone activity for the late twenty-first century from dynamical downscaling of CMIP5/RCP4.5 Scenarios. J Climate https://doi.org/10.1175/JCLI-D-15-0129.1, https://journals.ametsoc.org/view/journals/clim/28/18/jcli-d-15-0129.1.xml

[CR34] Knutson TR, Sirutis JJ, Bender MA, et al (2022) Dynamical downscaling projections of late twenty-first-century U.S. landfalling hurricane activity. Climatic Change 171(3):28. 10.1007/s10584-022-03346-7

[CR35] Langousis A, Veneziano D (2009) Theoretical model of rainfall in tropical cyclones for the assessment of long-term risk. J Geophys Res: Atmos 114(D2). 10.1029/2008JD010080, https://onlinelibrary.wiley.com/doi/abs/10.1029/2008JD010080, _eprint: https://agupubs.onlinelibrary.wiley.com/doi/pdf/10.1029/2008JD010080

[CR36] Lau K, Tam C, Wu C (2024) Island-induced eyewall replacement in a landfalling tropical cyclone: A model study of Super Typhoon Mangkhut (2018). J Geophys Res: Atmos 129(4):e2023JD039541. Publisher: Wiley Online Library

[CR37] Lavender SL, Mcbride JL (2021) Global climatology of rainfall rates and lifetime accumulated rainfall in tropical cyclones: Influence of cyclone basin, cyclone intensity and cyclone size. International Journal of Climatology 41(S1). 10.1002/joc.6763, type: Journal Article

[CR38] Lee CY, Tippett MK, Sobel AH et al (2018) An environmentally forced tropical cyclone hazard model. J Adv Modeling Earth Syst 10(1):223–241. 10.1002/2017MS001186.

[CR39] Lin YL, Chiao S, Wang TA et al (2001) Some common ingredients for heavy orographic rainfall. Weather Forecast 16(6):633–660

[CR40] Lin Y, Zhao M, Zhang M (2015) Tropical cyclone rainfall area controlled by relative sea surface temperature. Nat Commun 6(1):6591 25761457 10.1038/ncomms7591 PMC4382685

[CR41] Lin J, Rousseau-Rizzi R, Lee CY, et al (2023) An Open-Source, Physics-Based, Tropical Cyclone Downscaling Model With Intensity-Dependent Steering. Journal of Advances in Modeling Earth Systems 15(11):e2023MS003686. 10.1029/2023MS003686, https://onlinelibrary.wiley.com/doi/abs/10.1029/2023MS003686, _eprint: https://agupubs.onlinelibrary.wiley.com/doi/pdf/10.1029/2023MS003686

[CR42] Liu M, Vecchi GA, Smith JA, et al (2019) Causes of large projected increases in hurricane precipitation rates with global warming. npj Climate and Atmospheric Science 2(1):38. https://doi.org/10.1038/s41612-019-0095-3, https://www.nature.com/articles/s41612-019-0095-3, publisher: Nature Publishing Group

[CR43] Lonfat M, Marks FD Jr, Chen SS (2004) Precipitation distribution in tropical cyclones using the tropical rainfall measuring mission (TRMM) microwave imager: a global perspective. Mon Weather Rev 132(7):1645–1660

[CR44] Lonfat M, Rogers R, Marchok T et al (2007) A parametric model for predicting Hurricane rainfall. Mon Weather Rev. 10.1175/MWR3433.1.

[CR45] Lu P, Lin N, Emanuel K et al (2018) Assessing hurricane rainfall mechanisms using a physics-based model: Hurricanes Isabel (2003) and Irene (2011). J Atmos Sci. 10.1175/JAS-D-17-0264.1

[CR46] Marchok T, Rogers R, Tuleya R (2007) Validation schemes for tropical cyclone quantitative precipitation forecasts: evaluation of operational models for u.s. landfalling cases. Weather and Forecasting 10.1175/WAF1024.1, https://journals.ametsoc.org/view/journals/wefo/22/4/waf1024 _1.xml

[CR47] Matyas CJ (2014) Conditions associated with large rain-field areas for tropical cyclones landfalling over Florida. Phys Geogr 35(2):93–106. 10.1080/02723646.2014.893476

[CR48] Muñoz Sabater J (2019) ERA5-Land hourly data from 1950 to present. 10.24381/cds.e2161bac

[CR49] Nam CC, Park DSR, Ho CH et al (2018) Dependency of tropical cyclone risk on track in South Korea. Nat Hazard 18(12):3225–3234

[CR50] Park DSR, Ho CH, Nam CC et al (2015) Evidence of reduced vulnerability to tropical cyclones in the Republic of Korea. Environ Res Lett 10(5):054003

[CR51] Pérez-Alarcón A, Fernández-Alvarez JC, Coll-Hidalgo P (2023) Global increase of the intensity of tropical cyclones under global warming based on their maximum potential intensity and CMIP6 models. Environ Process 10(2):36. 10.1007/s40710-023-00649-4

[CR52] Rappaport EN (2014) Fatalities in the United States from Atlantic tropical cyclones: new data and interpretation. Bull Am Meteor Soc 95(3):341–346

[CR53] Scoccimarro E, Gualdi S, Villarini G et al (2014) Intense precipitation events associated with landfalling tropical cyclones in response to a warmer climate and increased CO2. J Clim. 10.1175/JCLI-D-14-00065.1

[CR54] Shepherd TG, Boyd E, Calel RA et al (2018) Storylines: an alternative approach to representing uncertainty in physical aspects of climate change. Clim Change 151(3):555–571. 10.1007/s10584-018-2317-9 30880852 10.1007/s10584-018-2317-9 PMC6394420

[CR55] Sobel AH, Lee CY, Bowen SG et al (2023) Near-term tropical cyclone risk and coupled Earth system model biases. Proc Natl Acad Sci 120(33):e2209631120. 10.1073/pnas.2209631120 37549274 10.1073/pnas.2209631120 PMC10438837

[CR56] Sparks N, Toumi R (2025) Climate change attribution of typhoon haiyan with the imperial college storm model. Atmos Sci Lett 26(1):e1285

[CR57] Sparks N, Toumi R (2024) The Imperial college storm model (IRIS) dataset. Scientific data 11(1):424. Publisher: Nature Publishing Group UK London 10.1038/s41597-024-03250-y PMC11043368 38658585

[CR58] Sparks N, Toumi R (2025) The impact of global warming on U.S. hurricane landfall: A storyline approach. Environmental Research Letters (under review)

[CR59] Stansfield AM, Reed KA, Zarzycki CM (2020) Changes in precipitation from north atlantic tropical cyclones under RCP scenarios in the variable-resolution community atmosphere model. Geophysical Research Letters 47(12):e2019GL086930. 10.1029/2019GL086930, https://onlinelibrary.wiley.com/doi/abs/10.1029/2019GL086930, _eprint:https://agupubs.onlinelibrary.wiley.com/doi/pdf/10.1029/2019GL086930

[CR60] Stern DP, Vigh JL, Nolan DS et al (2015) Revisiting the relationship between eyewall contraction and intensification. J Atmos Sci 72(4):1283–1306. 10.1175/JAS-D-14-0261.1

[CR61] Titley HA, Cloke HL, Harrigan S et al (2021) Key factors influencing the severity of fluvial flood hazard from tropical cyclones. J Hydrometeorol 22(7):1801–1817

[CR62] Toumi R, Sparks N (2025) The Hurricane Damage Index (HurDI). Journal of Catastrophe Risk and Resilience https://doi.org/10.63024/k2gm-2j0m, https://journalofcrr.com/research/03-05-toumi-sparks/

[CR63] Tsuboki K, Yoshioka MK, Shinoda T et al (2015) Future increase of supertyphoon intensity associated with climate change. Geophys Res Lett 42(2):646–652. 10.1002/2014GL061793

[CR64] Tu S, Xu J, Chan JC, et al (2021) Recent global decrease in the inner-core rain rate of tropical cyclones. Nature Communications 12(1):1948. ISBN: 2041-1723 Publisher: Nature Publishing Group UK London 10.1038/s41467-021-22304-y PMC8007805 33782405

[CR65] Tu S, Chan JC, Xu J, et al (2022) Increase in tropical cyclone rain rate with translation speed. Nature communications 13(1):7325. ISBN: 2041-1723 Publisher: Nature Publishing Group UK London 10.1038/s41467-022-35113-8 PMC9705401 36443318

[CR66] Tuleya RE, DeMaria M, Kuligowski RJ (2007) Evaluation of GFDL and simple statistical model rainfall forecasts for US landfalling tropical storms. Weather Forecast 22(1):56–70

[CR67] Ten Veldhuis J, Clemens F (2010) Flood risk modelling based on tangible and intangible urban flood damage quantification. Water Sci Technol 62(1):189–195 20595770 10.2166/wst.2010.243

[CR68] Vickery PJ, Skerlj PF, Twisdale LA (2000) Simulation of hurricane risk in the U.S. using empirical track model. J Struct Eng126(10):1222–1237. 10.1061/(ASCE)0733-9445(2000)126:10(1222), https://ascelibrary.org/doi/10.1061/%28ASCE%290733-9445%282000%29126%3A10%281222%29, publisher: American Society of Civil Engineers

[CR69] Villarini G, Lavers DA, Scoccimarro E et al (2014) Sensitivity of tropical cyclone rainfall to idealized global-scale forcings. J Clim. 10.1175/JCLI-D-13-00780.1

[CR70] Wang S, Toumi R (2022) On the intensity decay of tropical cyclones before landfall. Scientific reports 12(1):3288. Publisher: Nature Publishing Group UK London 10.1038/s41598-022-07310-4 PMC8885634 35228600

[CR71] Wehner M, Prabhat, Reed KA, et al (2015) Resolution dependence of future tropical cyclone projections of CAM5.1 in the U.S. CLIVAR hurricane working group idealized configurations. J Clim 28(10):3905–3925. https://doi.org/10.1175/JCLI-D-14-00311.1, https://journals.ametsoc.org/view/journals/clim/28/10/jcli-d-14-00311.1.xml

[CR72] Wen S, Su B, Wang Y et al (2018) Economic sector loss from influential tropical cyclones and relationship to associated rainfall and wind speed in China. Global Planet Change 169:224–233

[CR73] Willoughby H, Clos J, Shoreibah M (1982) Concentric eye walls, secondary wind maxima, and the evolution of the hurricane vortex. J Atmos Sci 39(2):395–411

[CR74] Wright DB, Knutson TR, Smith JA (2015) Regional climate model projections of rainfall from U.S. landfalling tropical cyclones. Climate Dynamics 45(11):3365–3379. 10.1007/s00382-015-2544-y,

[CR75] Wu C, Chou K, Cheng H, et al (2003) Eyewall contraction, breakdown and reformation in a landfalling typhoon. Geophysical research letters 30(17). Publisher: Wiley Online Library

[CR76] Yamada Y, Satoh M, Sugi M et al (2017) Response of tropical cyclone activity and structure to global warming in a high-resolution global nonhydrostatic model. J Clim 30(23):9703–9724

[CR77] Yang Y, Toumi R (2025) Large dynamic contributions to tropical cyclone precipitation with increasing sea surface temperature. Environ Res Lett 20(7):074013. 10.1088/1748-9326/add753

[CR78] Yoshida K, Sugi M, Mizuta R et al (2017) Future changes in tropical cyclone activity in high-resolution large-ensemble simulations. Geophys Res Lett 44(19):9910–9917. 10.1002/2017GL075058

[CR79] Zhong Q, Chan JCL, Duan W et al (2026) Landfalling tropical cyclones accelerate due to land–sea thermal and roughness contrasts. Nat Geosci 19(2):159–164. 10.1038/s41561-025-01891-1

[CR80] Zhu L, Quiring SM, Emanuel KA (2013) Estimating tropical cyclone precipitation risk in Texas. Geophys Res Lett 40(23):6225–6230. 10.1002/2013GL058284

