# Supplementary material for: A global parametric rain model for landfalling tropical cyclones: a case study for the U.S
Source: Nat Hazards (Dordr). 2026 Apr 29;122(10):415. doi: 10.1007/s11069-026-08150-5 (PMC13128782; doi:10.1007/s11069-026-08150-5)
Supplement: Supplementary file 1 — (pdf 8126 KB) [file 11069_2026_8150_MOESM1_ESM.pdf]

Supporting Information for

A global parametric rain model for landfalling tropical cyclones: A case study for the U.S.

King Heng Lau[1], Sacha Czernichow[1,2], Nathan Sparks[1], Ralf Toumi[1]

Corresponding Author: King Heng Lau (khlau@imperial.ac.uk)

Affiliations:

[1] Department of Physics, Imperial College London, London, SW7 2AZ, United Kingdom

[2] Université Paris-Saclay, ENS Paris-Saclay, 91190, Gif-sur-Yvette, France

Introduction

This document contains supplementary information for the main article:

- **Supplementary Figures S1 to S15**
- **Supplementary Tables S1 to S3**
- **Supplementary Text S1**

Supplementary Figures

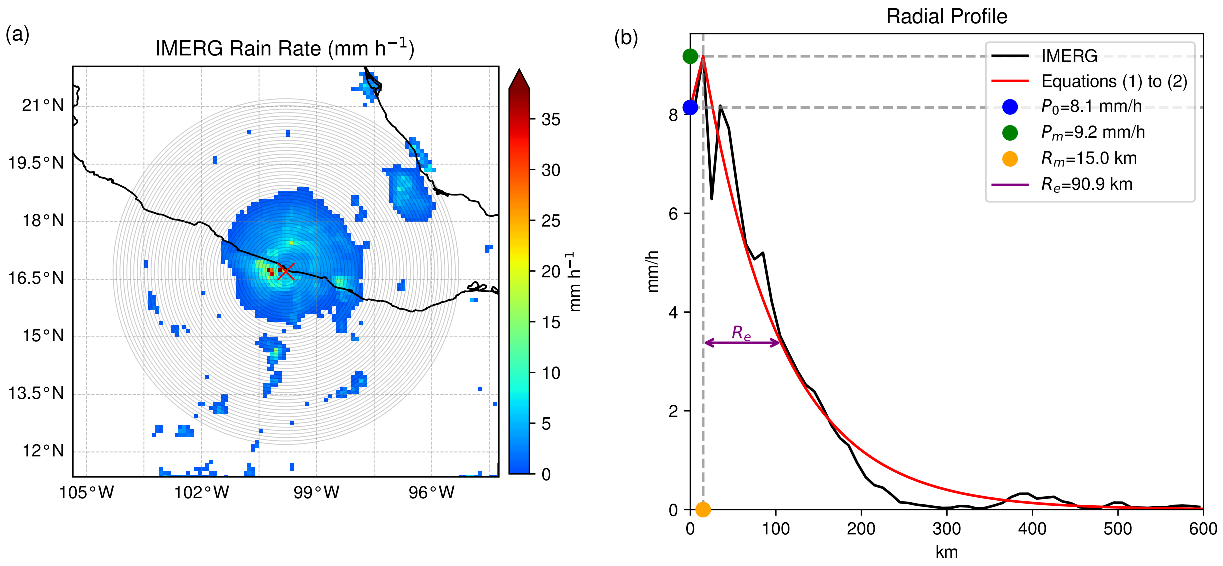

Supplementary Figure S1: An illustration of parameter calculation of the pre-landfall rain model (Equations (1)–(2) in the main text). (a) IMERG rain rate (shaded; mm h^{-1}) of Major Hurricane Otis (IBTrACS SID: 2023294N09264) at 2023-10-25 06:00 UTC, just before its landfall over Mexico (coastlines shown as black lines). Red cross shows the best-track TC centre and grey concentric circles up to 500 km from the TC centre illustrate the 10-km wide annuli used for azimuthal averaging of the rain field. (b) Radial profile of the azimuthally averaged rain rate (black line; mm h^{-1}) of Major Hurricane Otis at 2023-10-25 06:00 UTC. From the radial profile, P_0 (blue dot; mm h^{-1}), P_m (green dot; mm h^{-1}), R_m (orange dot; km) can be identified. Equation (1) in the main text was used to fit a linear function from the TC centre to $r = R_m$ (red line; mm h^{-1}). Equation (2) in the main text was used to fit an exponential decay function from $r = R_m$ to $r = 1000$km (red line; mm h^{-1}), which yields the e-folding radius R_e (purple double headed arrow; km).

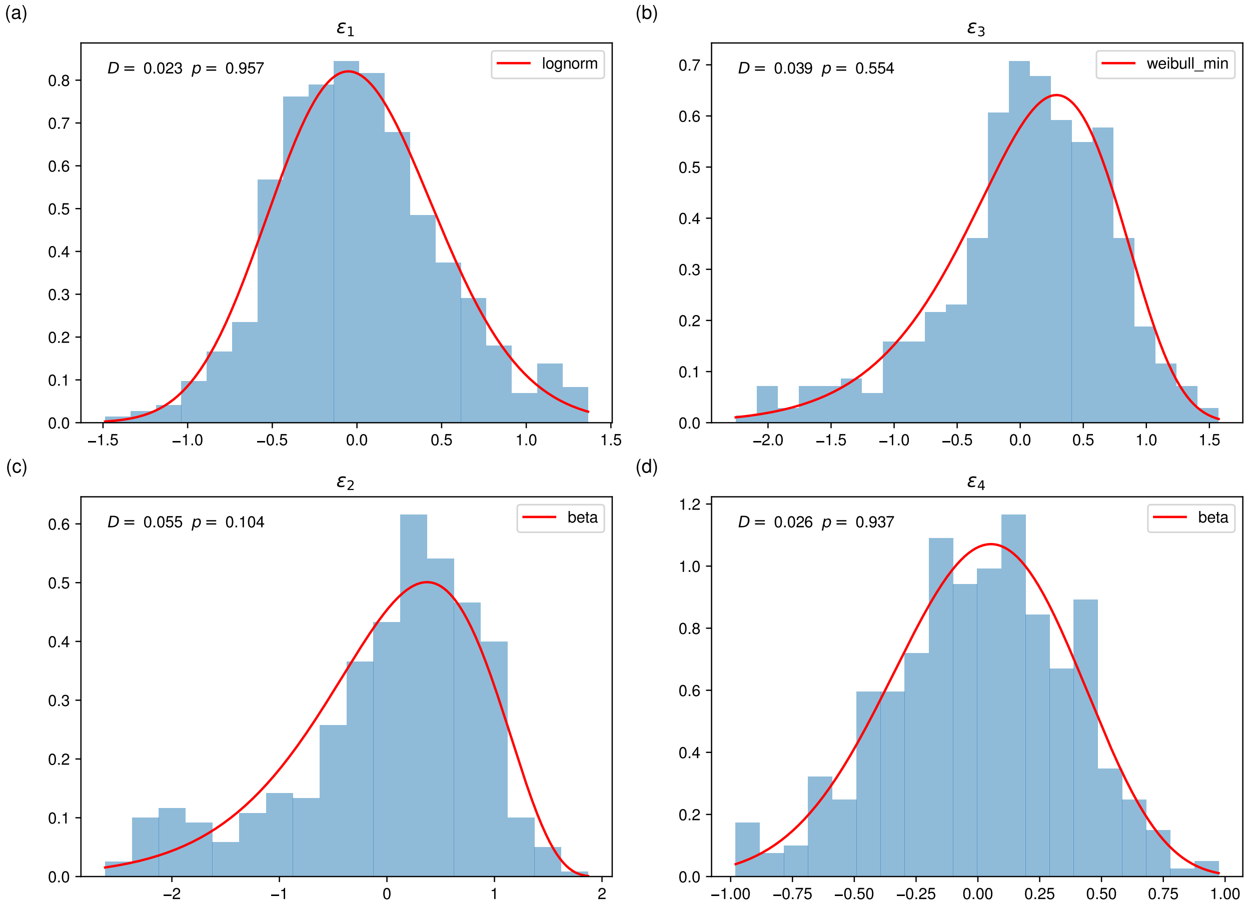

Supplementary Figure S2: The noise distributions used in the pre-landfall parametric TC rain model. Bars show the probability density of (a) ϵ_1, (b) ϵ_2, (c) ϵ_3, and (d) ϵ_4. Red curves show the fitted continuous distribution. The test statistic (D) and p-value (p) of the two-sample Kolmogorov-Smirnov test for goodness of fit are quoted. The null hypothesis is that the two distributions are identical.

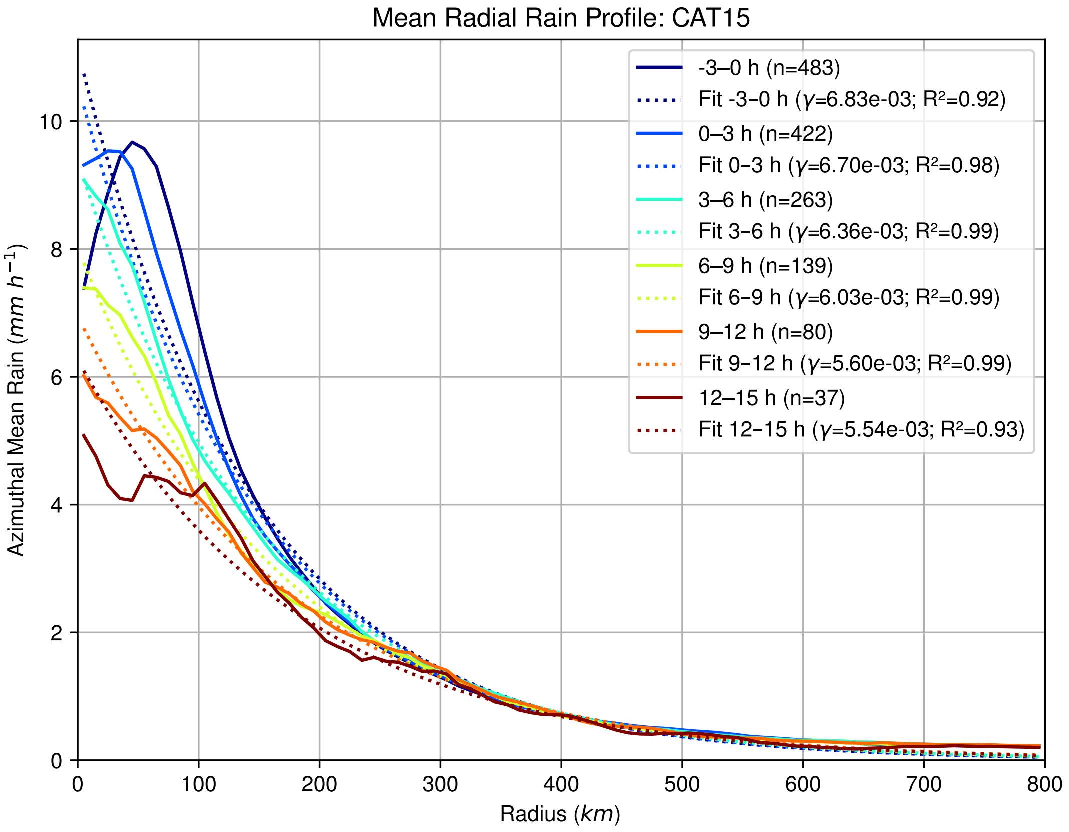

Supplementary Figure S3: Temporal evolution of the average radial profile of azimuthal mean rain rate (solid lines; $\mathrm{mm\,h^{-1}}$) for CAT1+ TCs. Legend shows groups by hours from landfall (positive for after landfall) with sample size (n) indicated. For each curve, an exponential decay function was fitted (dotted lines; $\mathrm{mm\,h^{-1}}$), with the radial decay constant (γ; $\mathrm{km^{-1}}$) and the coefficient of determination (R^2) displayed in the legend.

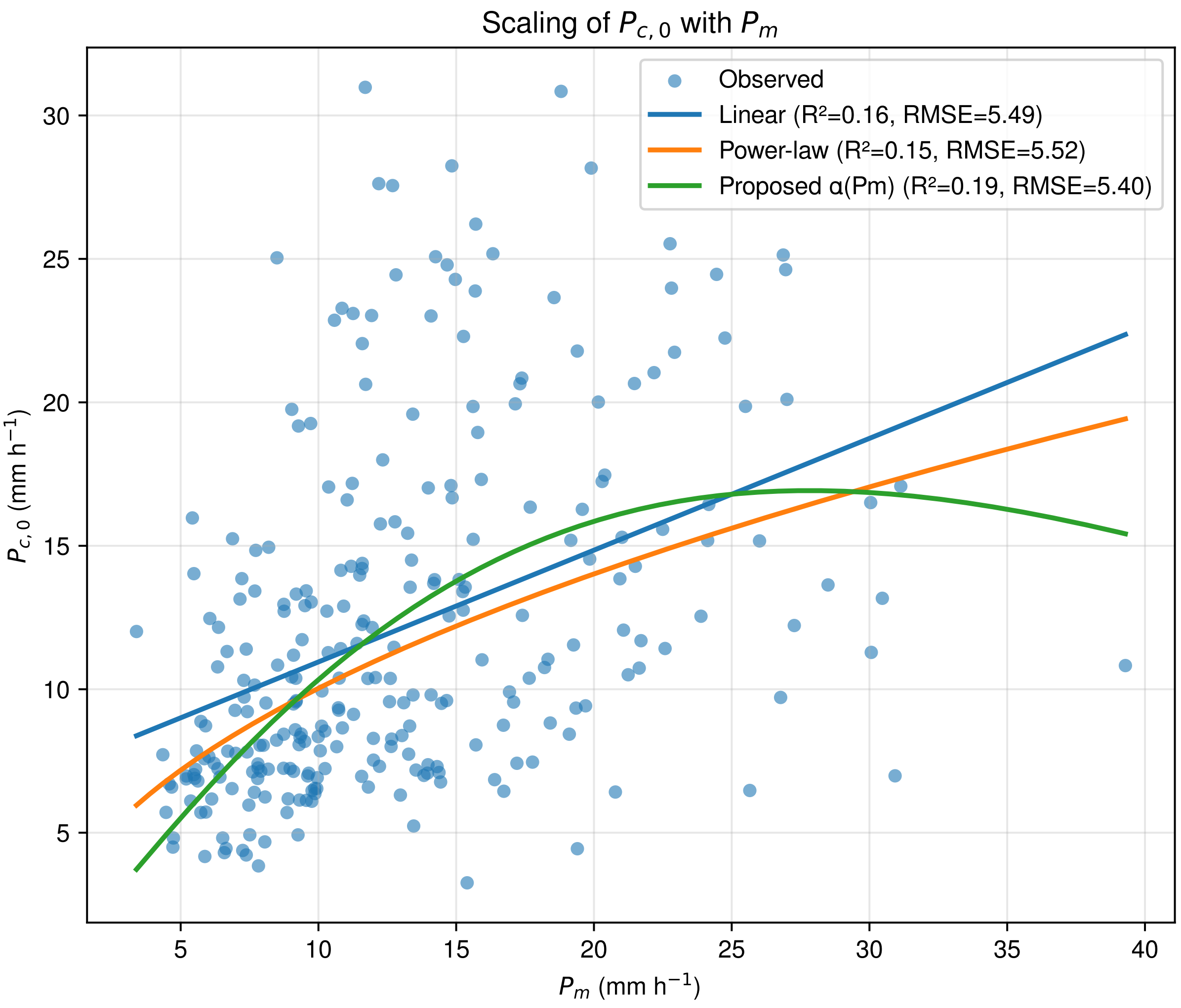

Supplementary Figure S4: Scatter plot of observed maximum azimuthally averaged rain rate (mm h^{-1}) over the first land point ($P_{c,0}$) versus the last ocean point (P_m) for CAT1+ landfall episodes used to fit the post-landfall rain model. Overlaid are a linear fit ($P_{c,0} = aP_m + b$, blue line), a power-law fit with constant exponent ($P_{c,0} = P_m^c$, orange line), and the exponential form model (Equation 8 in the main text; $P_{c,0} = P_m^{\alpha}$, green line) with α predicted from Equation 13. The mean value of ε_5 is used for clarity. The legend reports the goodness-of-fit for each model, including the coefficient of determination (R^2) and root-mean-squared error (RMSE).

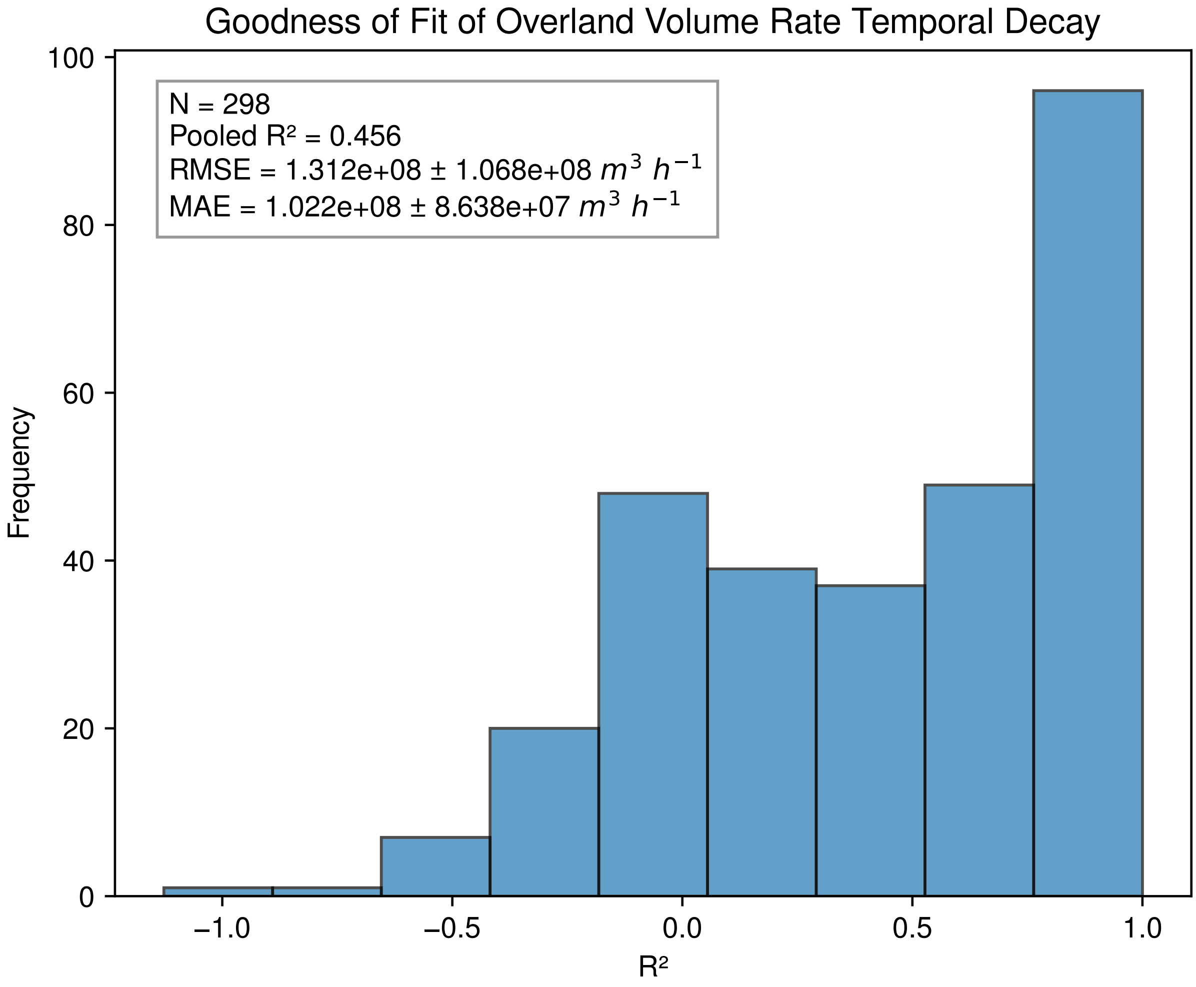

Supplementary Figure S5: Goodness of fit of the overland volume rate temporal decay. Bars show the distribution of the coefficient of determination (R^2). The sample size (N) and goodness-of-fit metrics, including the pooled R^2, root mean square error (RMSE), and mean absolute error (MAE), are quoted. For the RMSE and MAE, the standard deviation is shown.

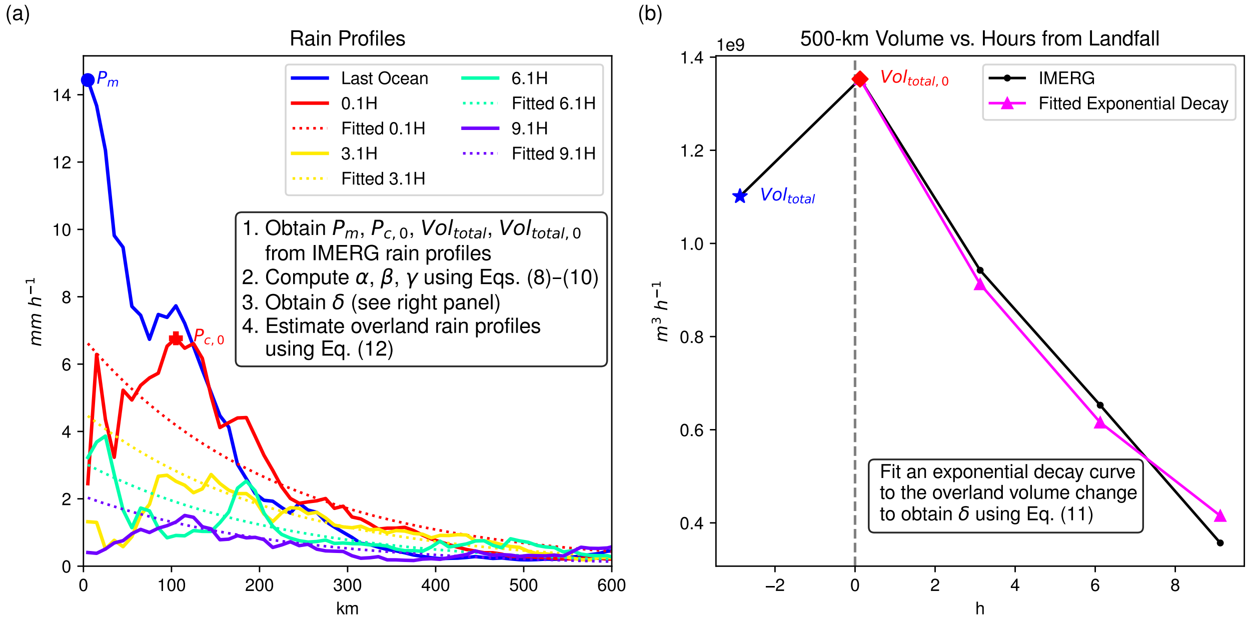

Supplementary Figure S6: An illustration of parameter calculation of the post-landfall rain model (Equations (7)–(12) in the main text). (a) Radial profiles of azimuthally averaged IMERG rain rate (solid lines; $\mathrm{mm\,h^{-1}}$) of Major Hurricane Lidia (IBTrACS SID: 2023276N11254) from its last ocean time step before landfall (2023-10-10 21:00 UTC) to its last overland time step (2023-10-11 09:00 UTC) at 3-hourly time intervals. For each of the four overland time steps, an exponential decay function (dashed lines; $\mathrm{mm\,h^{-1}}$) was fitted (Equation (7) of the main text). The value of the variables P_m (blue marker; $\mathrm{mm\,h^{-1}}$) and $P_{c,0}$ (red marker; $\mathrm{mm\,h^{-1}}$) are marked. Numbers in the legend indicate time elapsed (hours) after landfall. Procedures to estimate the four post-landfall rain parameters (i.e., α, β, γ, and δ) are described in text. (b) Temporal evolution of the IMERG 500-km rain volume rate (black line; $\mathrm{m^3\,h^{-1}}$) of Major Hurricane Lidia from its last ocean time step before landfall to its last overland time step at 3-hourly time intervals. For the overland evolution, an exponential decay function (magenta line; $\mathrm{m^3\,h^{-1}}$) was fitted (Equation (11) of the main text), from which the parameter δ was obtained. The value of the variables Vol_{total} (blue marker; $\mathrm{m^3\,h^{-1}}$) and $Vol_{total,0}$ (red marker; $\mathrm{m^3\,h^{-1}}$) are marked.

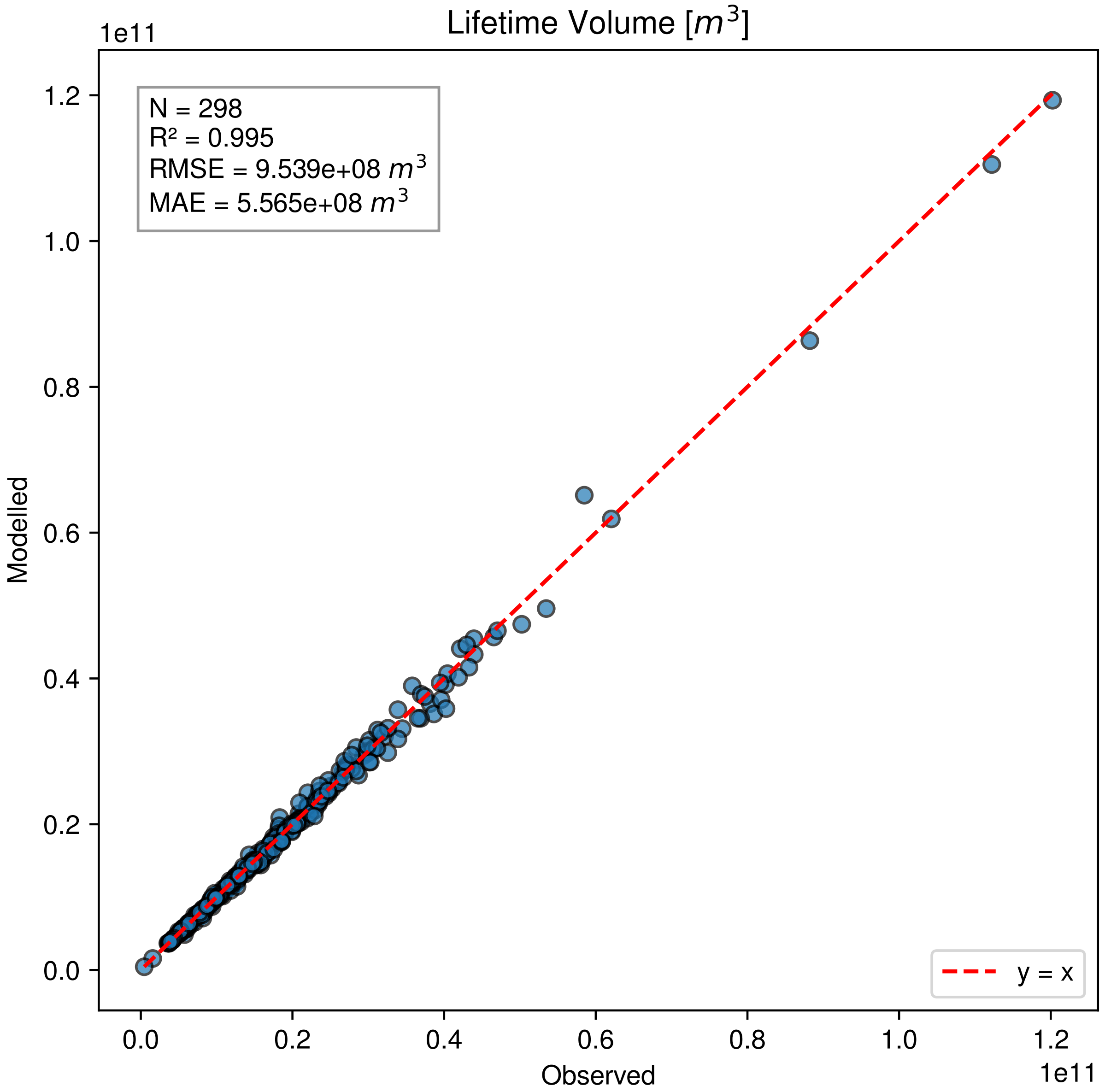

Supplementary Figure S7: Modelled post-landfall lifetime rain volume productions versus the observed for CAT1+ landfalls. Individual events are shown by filled circles. The line of equality is shown by a red dashed line. The sample size (N) and goodness-of-fit metrics are quoted as in Supplementary Figure S5.

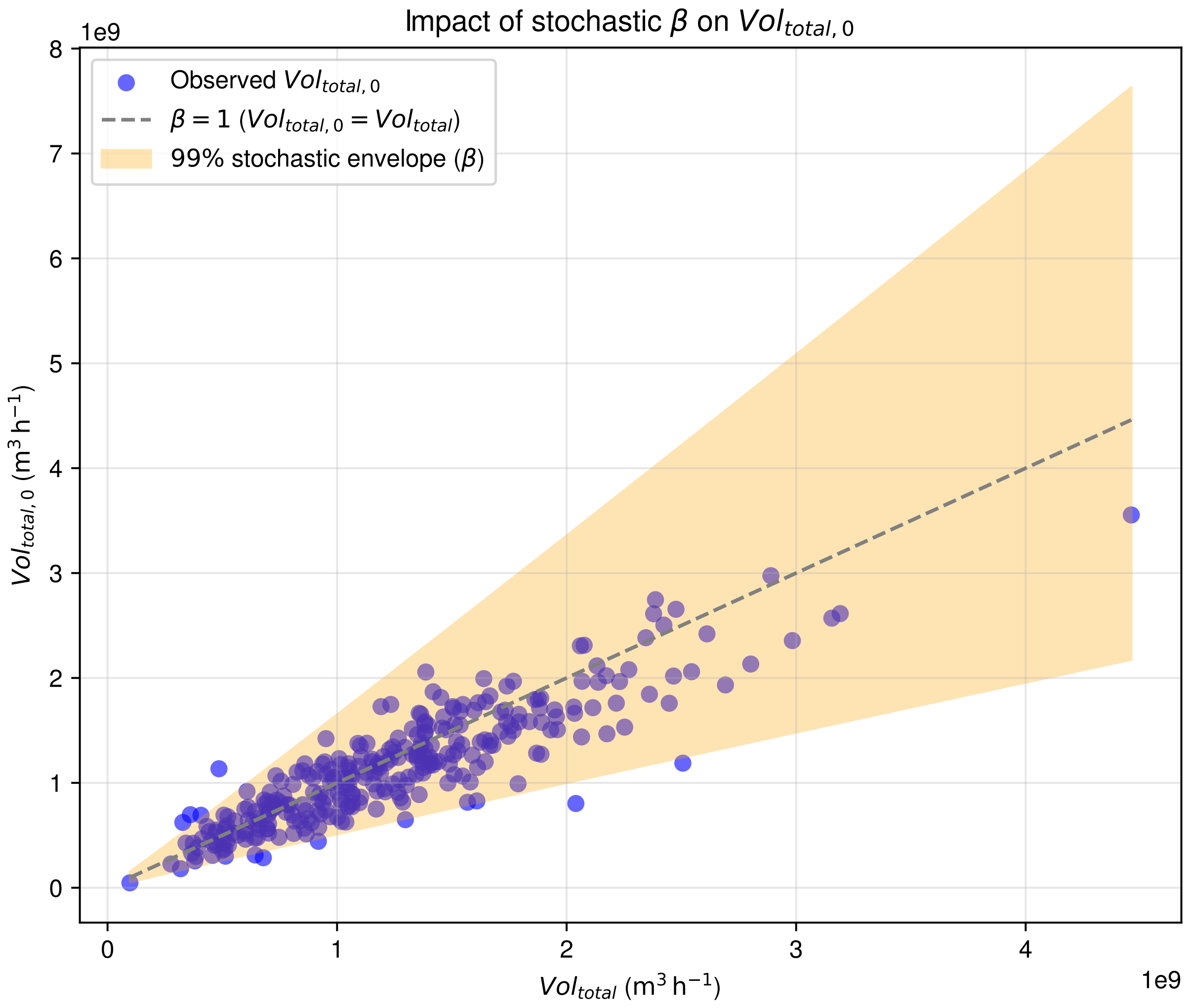

Supplementary Figure S8: Scatter plot of observed storm-total rain volume rate ($m^3\,h^{-1}$) over the first land point ($Vol_{total,0}$) versus the last ocean point (Vol_{total}) for CAT1+ landfall episodes used to fit the post-landfall rain model. The gray dashed line shows the 1:1 reference ($\beta = 1$). The orange shaded area represents the 99 % stochastic envelope of $Vol_{total,0}$ generated by sampling β from its fitted normal distribution (Equation 15 in the main text), illustrating the natural variability captured by the model.

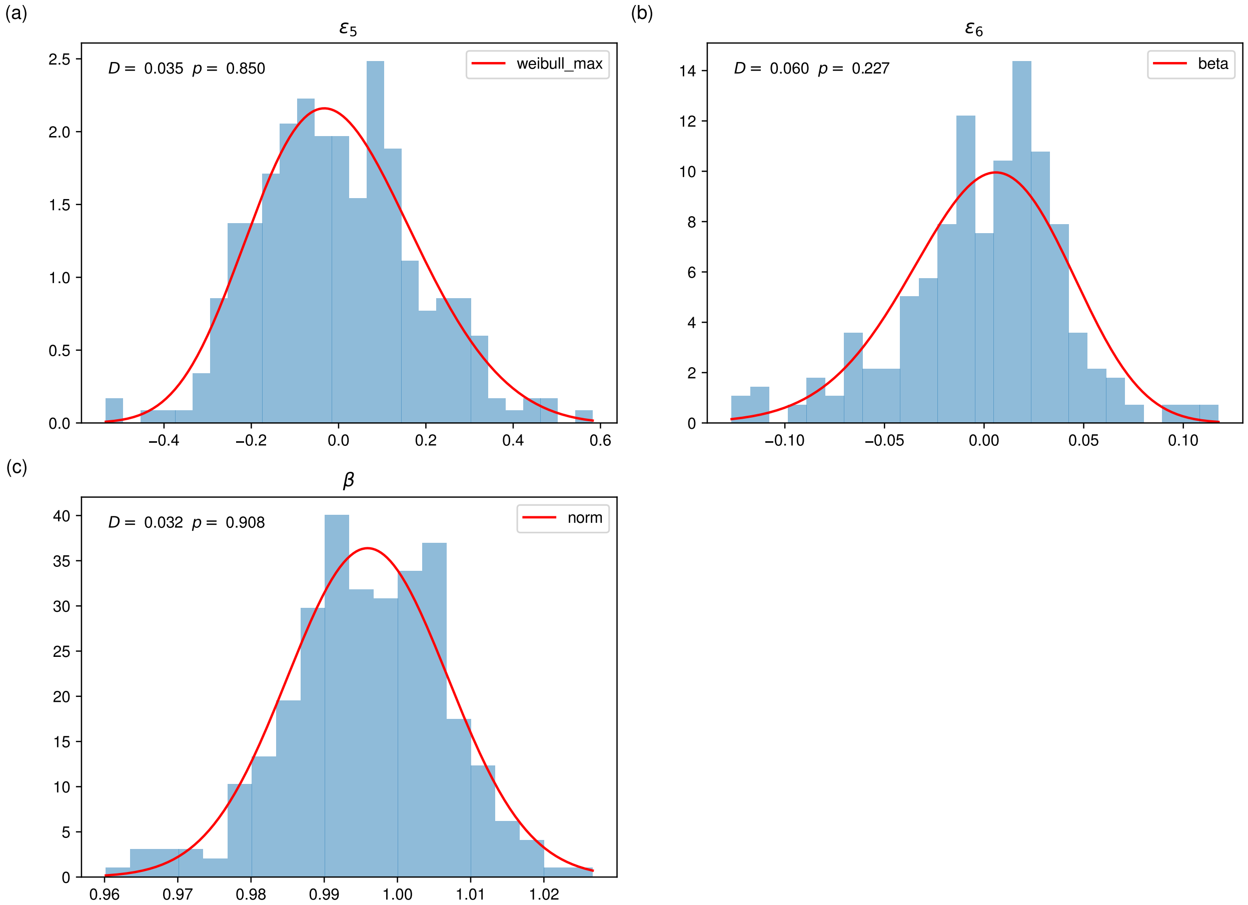

Supplementary Figure S9: The noise distributions used in the post-landfall parametric TC rain model and the sampling distribution of β. Bars show the probability density of (a) ϵ_5, (b) ϵ_6, and (c) β. Elements as in Supplementary Figure S2.

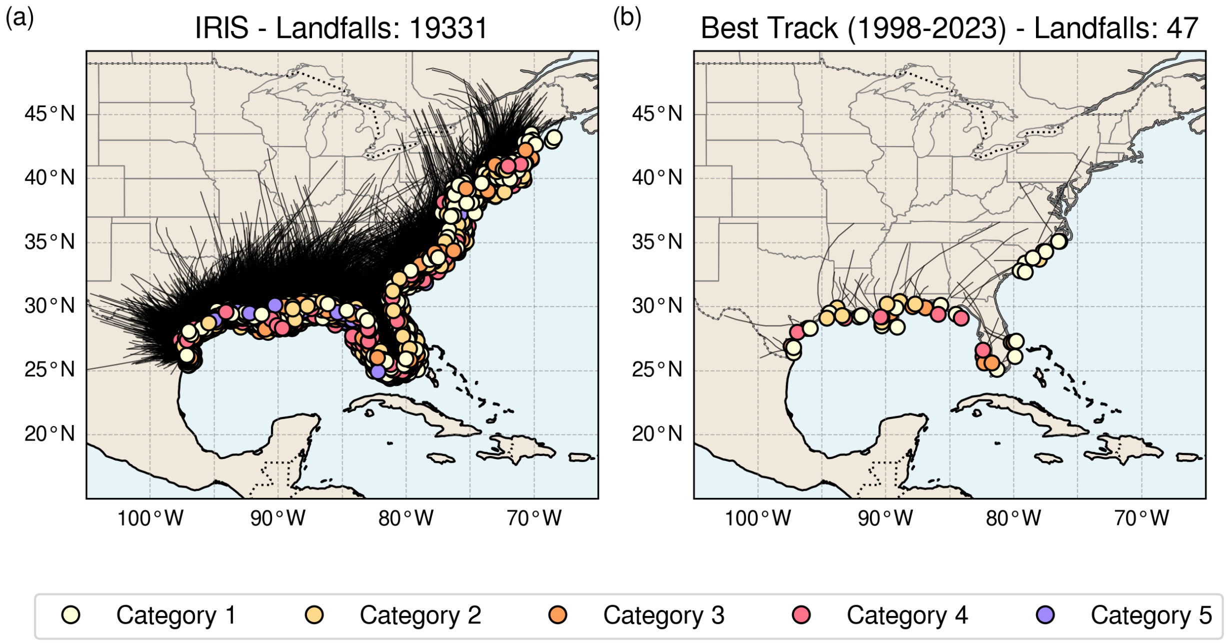

Supplementary Figure S10: Tracks of (a) stochastic TCs in the IRIS control run and (b) observed TCs in IBTrACS within the analysis period of 1998–2023 making landfalls over the U.S. with CAT1+ intensity. Filled circles denote the last ocean points prior to landfall, with colours indicating the SSHS category as shown in the legend.

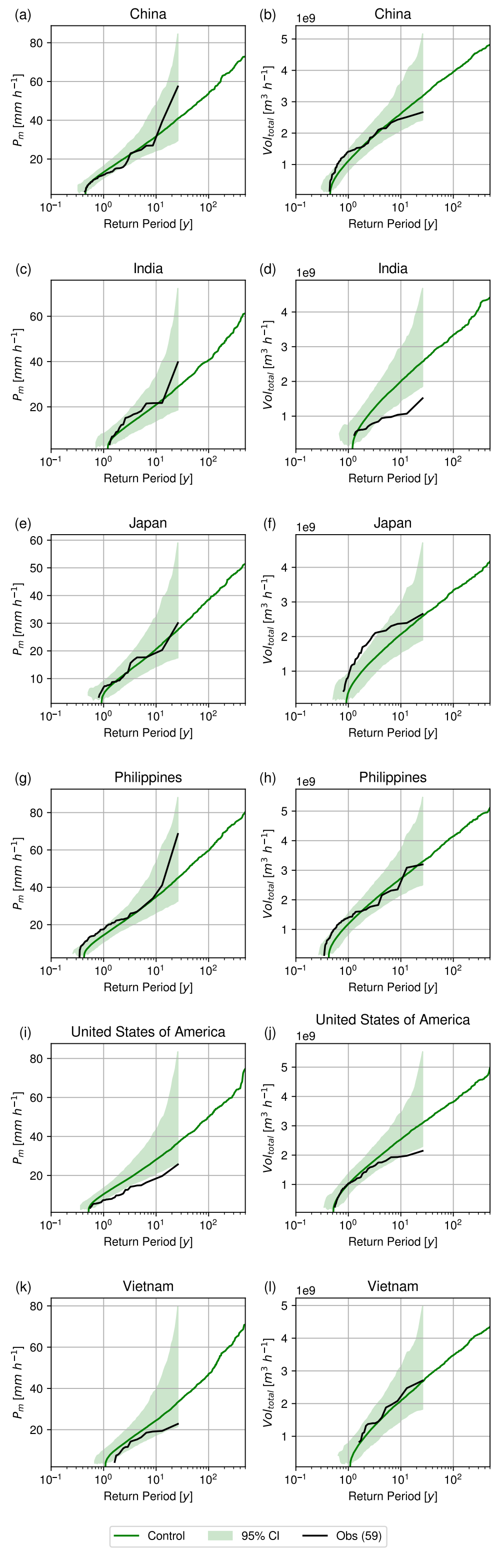

Supplementary Figure S11: As in Figure 2 in the main text, but for different countries: China ((a)–(b)), India ((c)–(d)), Japan ((e)–(f)), the Philippines ((g)–(h)), the U.S. ((i)–(j)), and Vietnam ((k)–(l)).

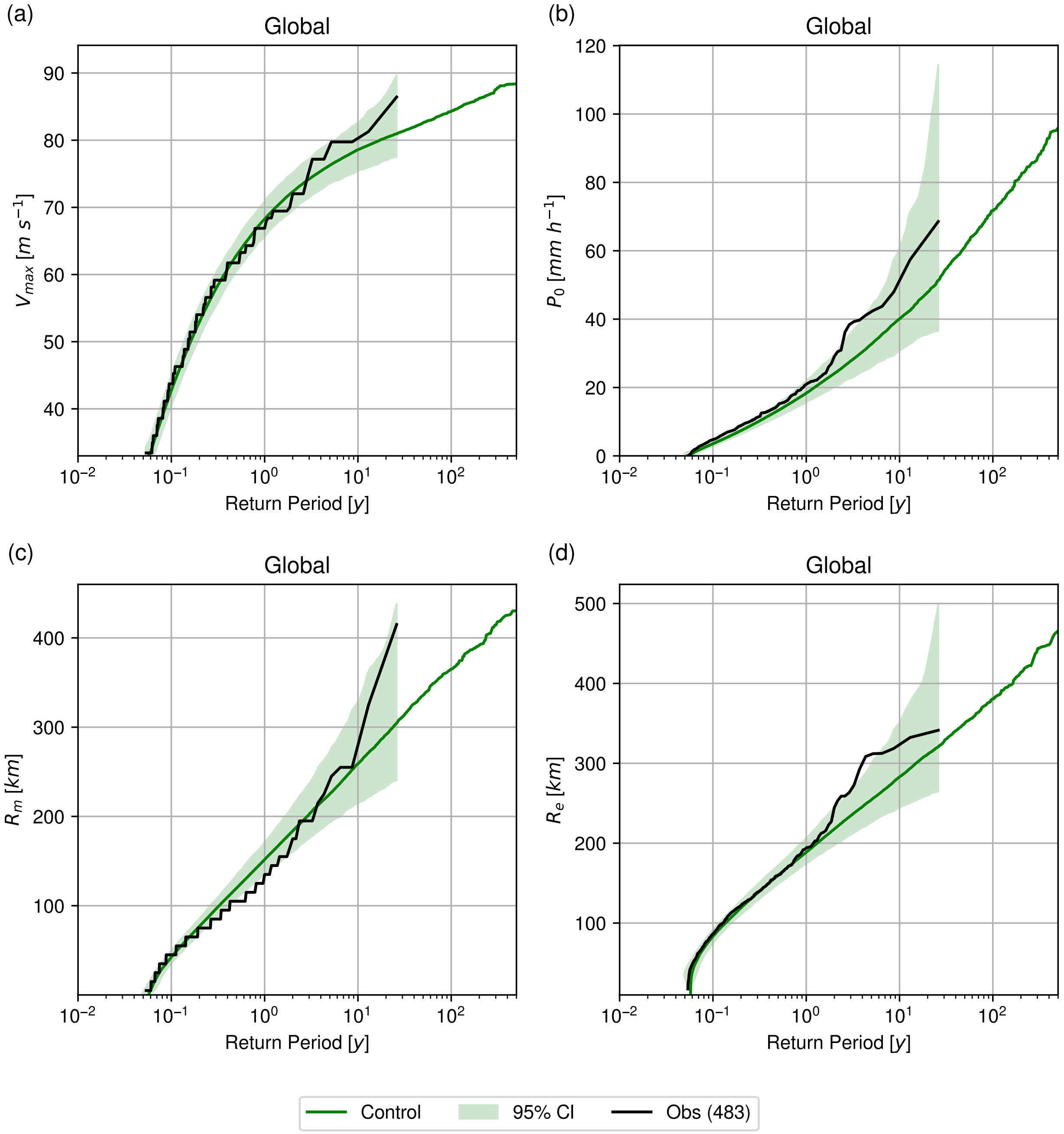

Supplementary Figure S12: Global validation of the IRIS model for the (a) V_{max} ($\mathrm{m\,s^{-1}}$), (b) P_0 ($\mathrm{mm\,h^{-1}}$), (c) R_m ($\mathrm{mm\,h^{-1}}$), and (d) R_e (km) at the last ocean point before landfall. Shown are return period curves from the 10 000-year IRIS control run (green) and from observations for 1998–2023 (black, with the number of events indicated in parentheses in the legend). The green shading indicates the 95 % confidence interval, estimated from bootstrapping 1000 26-year samples of the IRIS control run to match the observational record length.

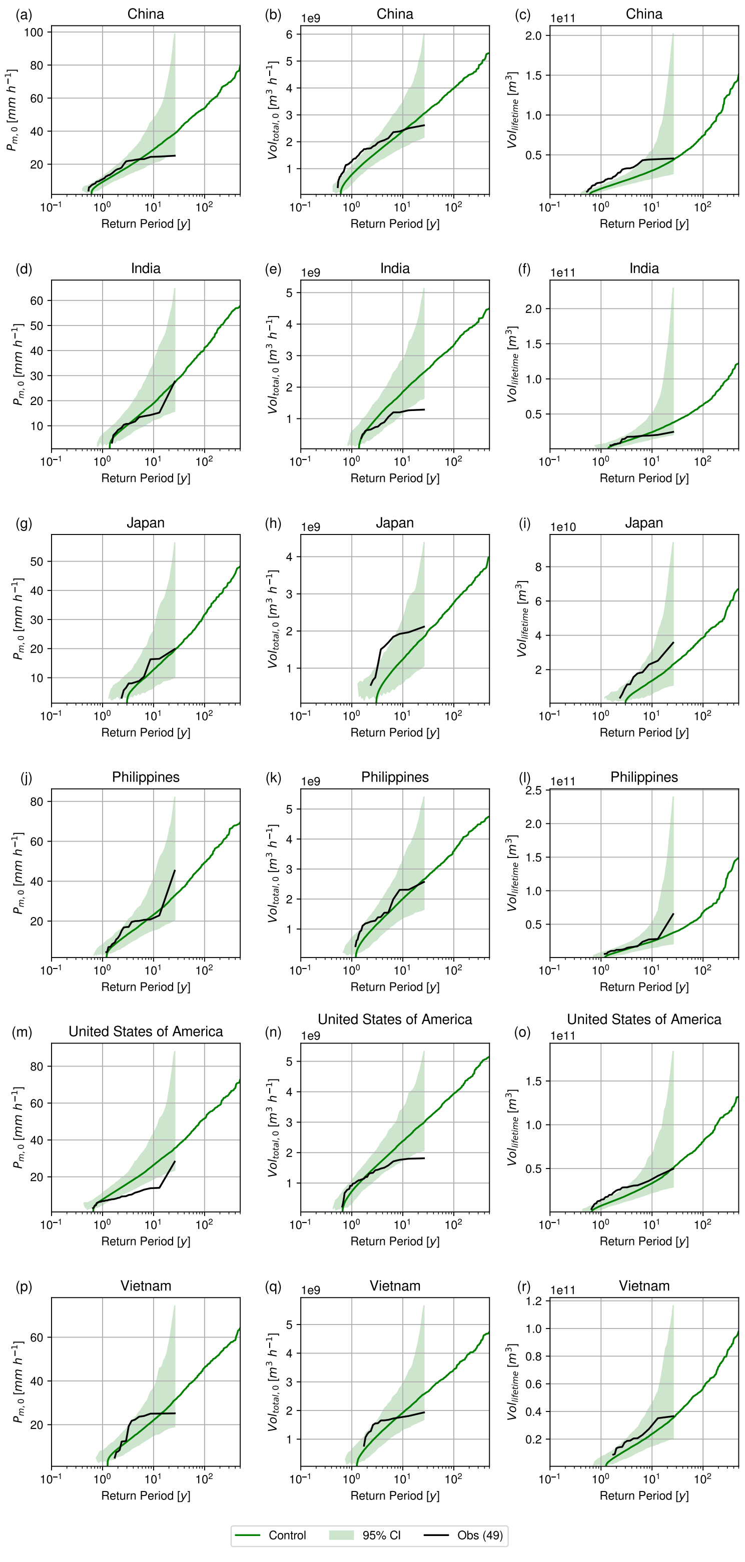

Supplementary Figure S13: As in Figure 3 in the main text, but for different countries: China ((a)–(c)), India ((d)–(f)), Japan ((g)–(i)), the Philippines ((j)–(l)), the U.S. ((m)–(o)), and Vietnam ((p)–(r)).

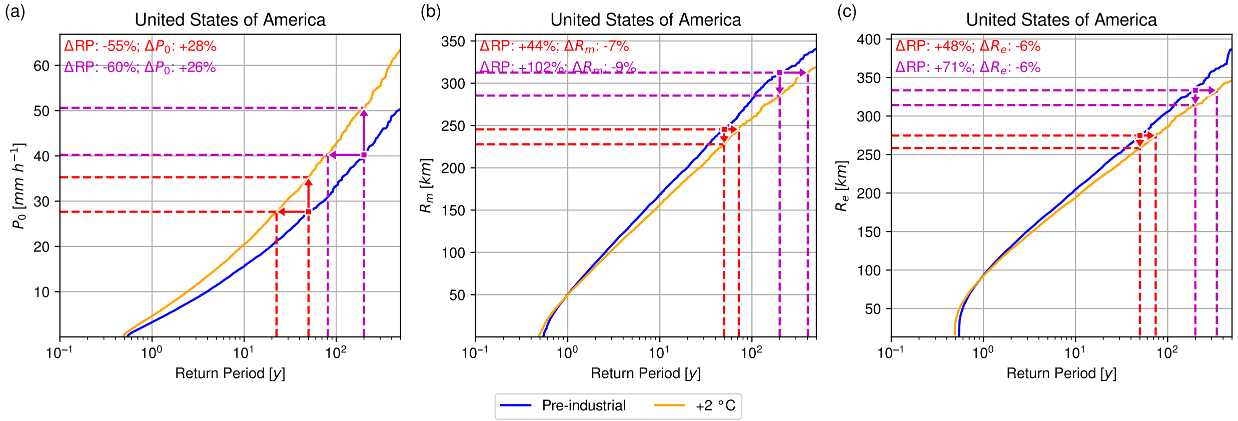

Supplementary Figure S14: Return curves for IRIS simulations of pre-industrial (blue) and +2 °C (orange) simulations for (a) P_0 (mm h^{-1}), (b) R_m (km), and (c) R_e (km) of U.S. landfalling hurricanes. Red (Magenta) lines and arrows show the impact of +2 °C on a 50- (200-) year event.

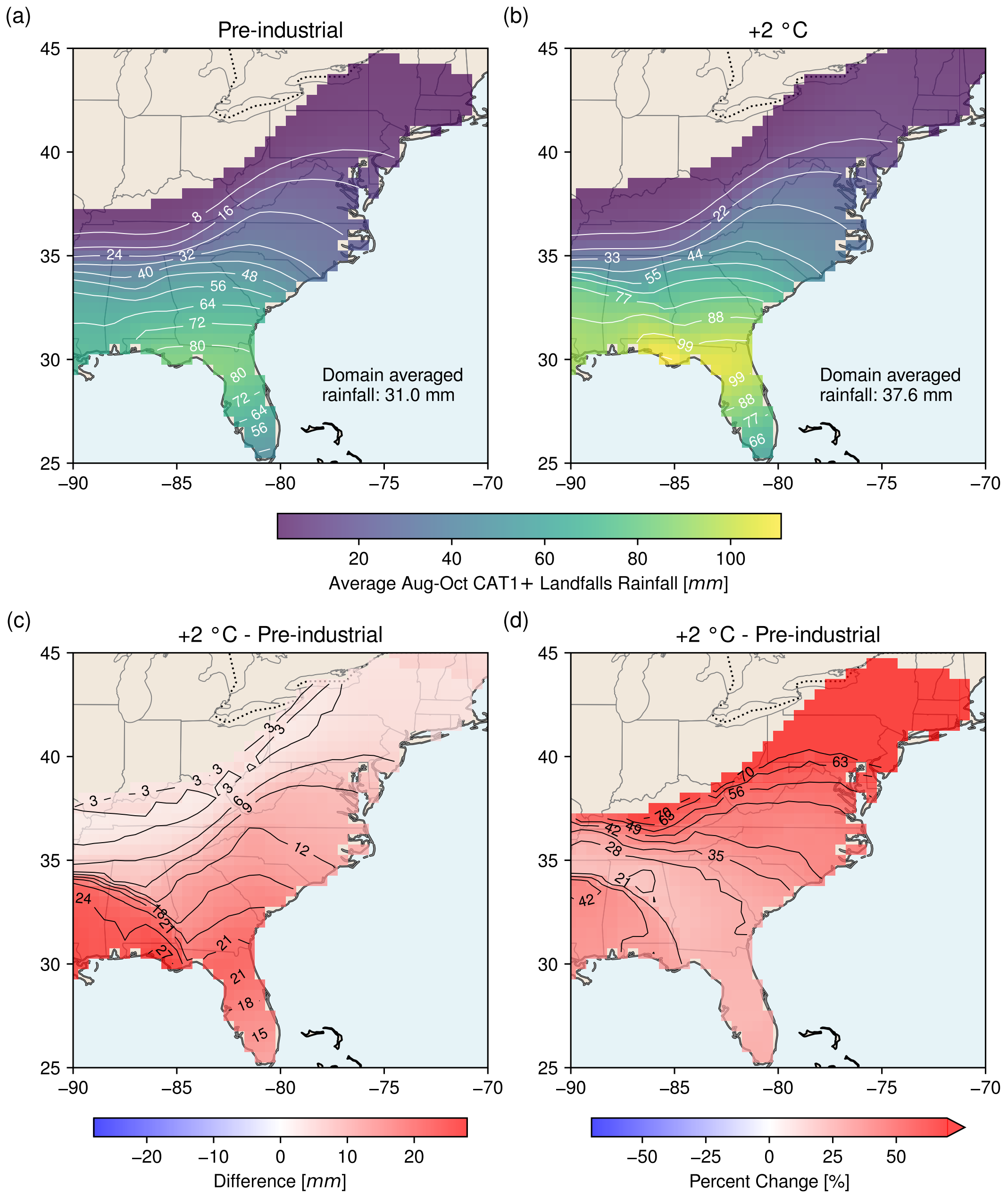

Supplementary Figure S15: Average August–October TC rainfall (mm) maps over the U.S. for (a) the pre-industrial scenario and (b) the +2 °C scenario. Panel (c) shows the difference between the two scenarios (+2 °C minus pre-industrial), and panel (d) the corresponding percentage change. Areas in which rainfall is less than 2.5 mm are excluded for clarity. Plotted for comparison with Figure 4 of Wright et al. (2015).

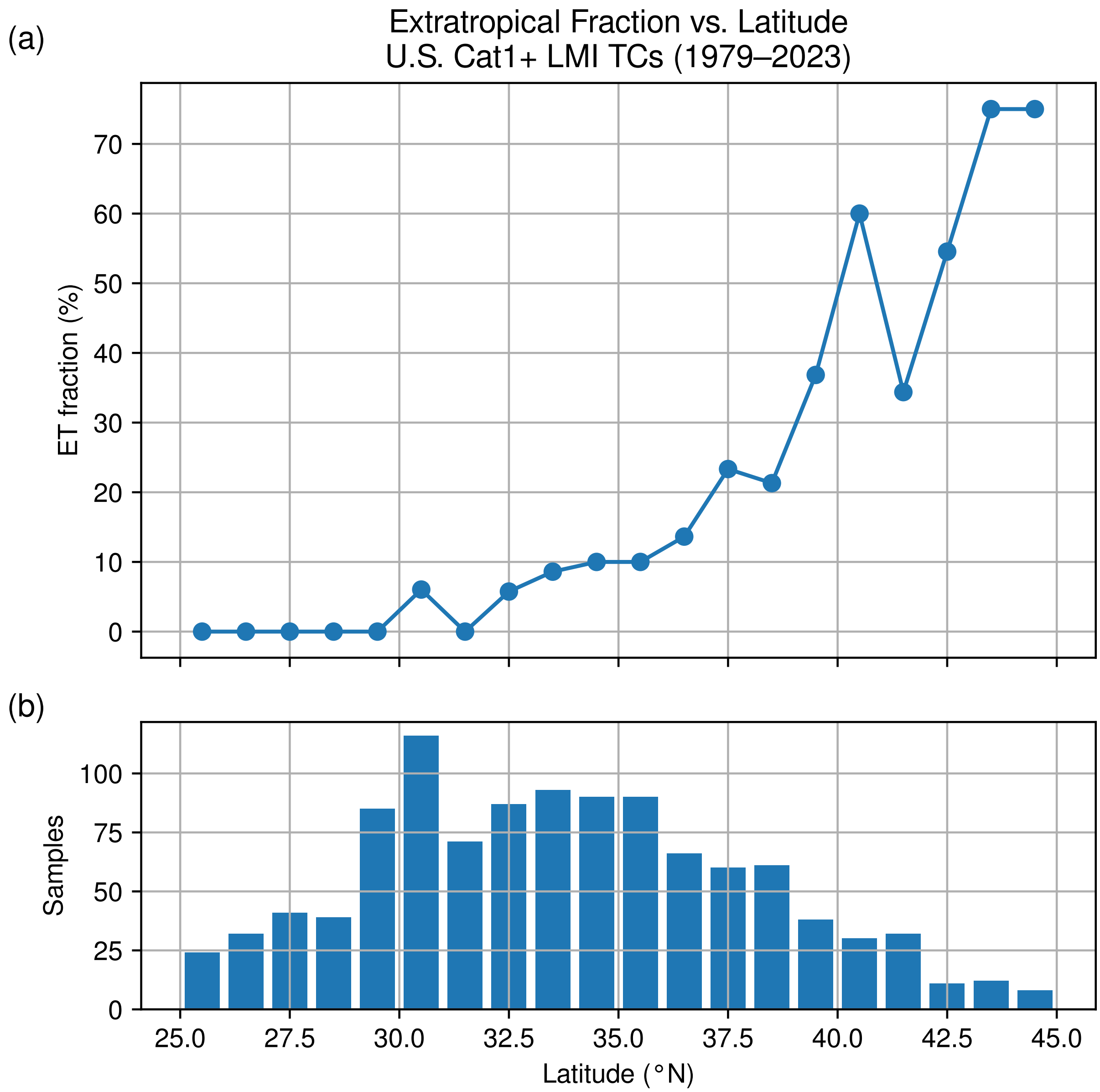

Supplementary Figure S16: (a) Fraction (%) and (b) Number of IBTrACS instantaneous records classified as "Extratropical" in the "NATURE" flag in IBTrACS as a function of latitude (° N) over the continental U.S.. All tracks with a CAT1+ LMI within the period 1979–2023 are included.

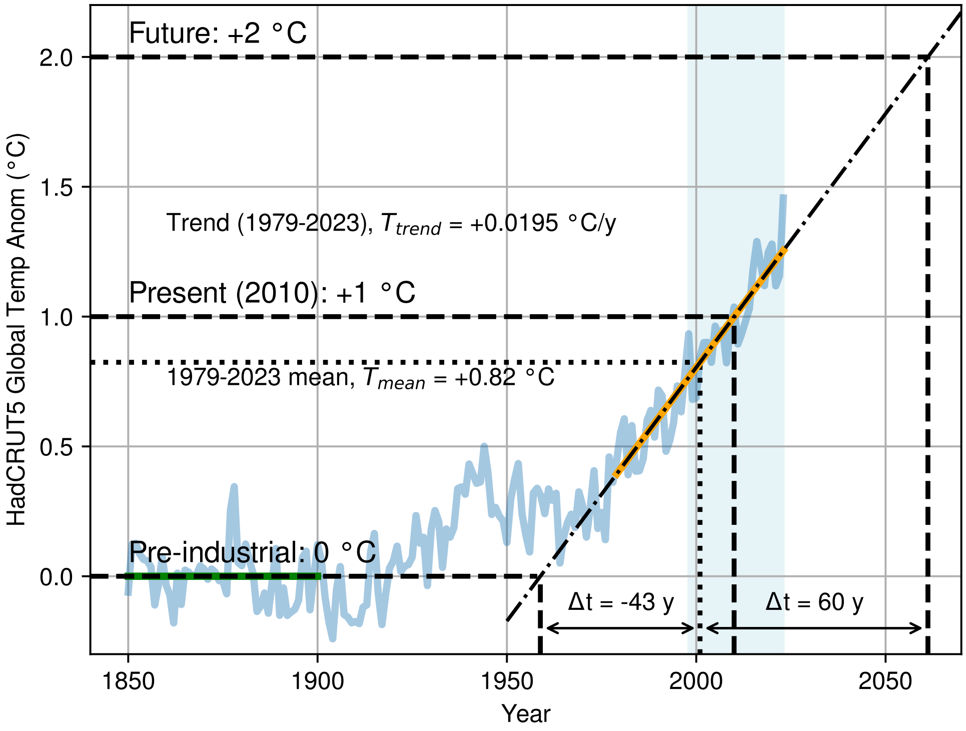

Supplementary Figure S17: Global mean surface temperature (°C) illustrating the scaling method. The HadCRUT5 global mean surface temperature anomaly is shown as a blue solid line. The trend line regressed to 1979–2023 is shown as a black dash-dotted line. The mean anomaly for the same period is indicated by dotted lines. The pre-industrial, present, and +2 °C scenarios are indicated by dashed lines. The number of years used to scale the mean anomaly to the pre-industrial and +2 °C scenarios are shown as arrows.

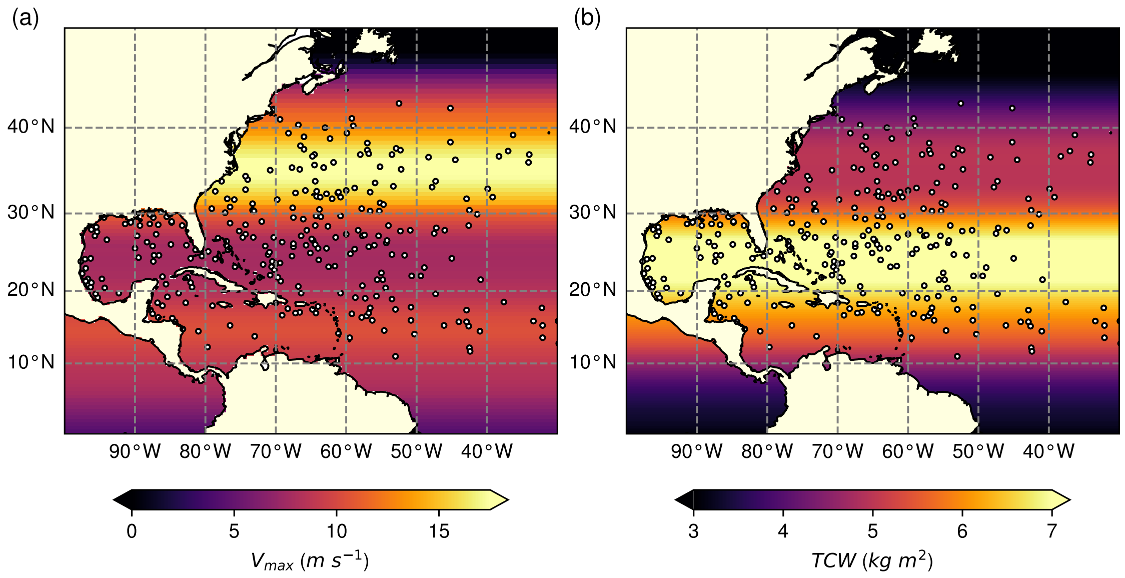

Supplementary Figure S18: Change in global zonally averaged September PI and TCW from pre-industrial to +2 °C in the NA applied in IRIS. Dots show the locations of the LMIs of observed September CAT1+ TCs since 1900.

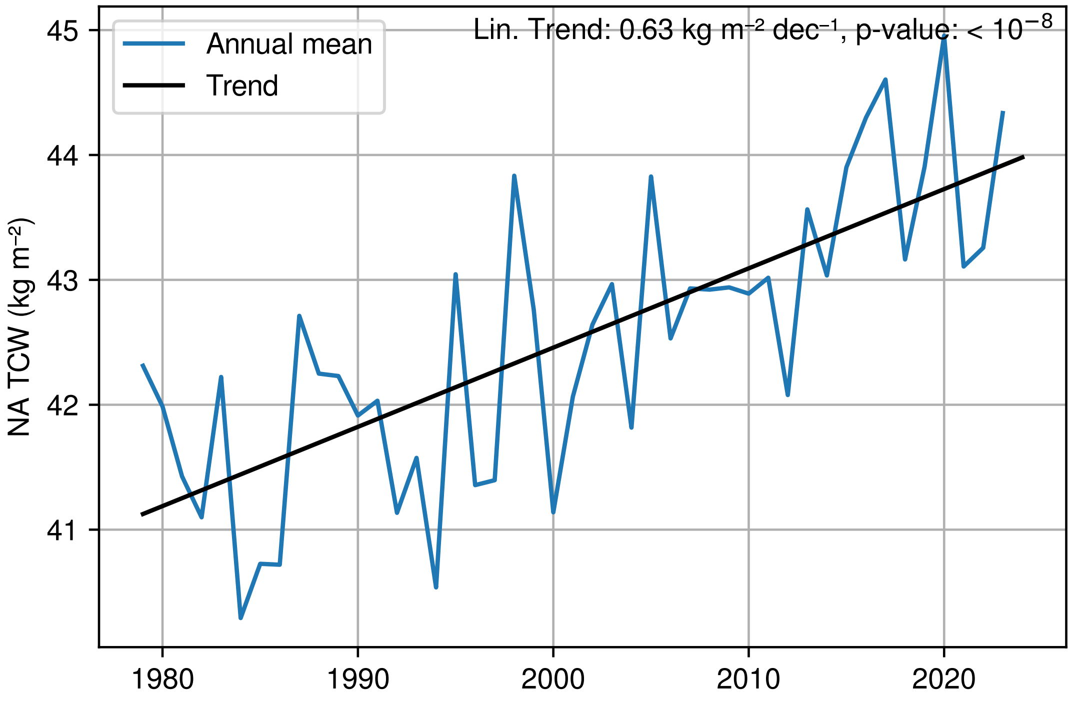

Supplementary Figure S19: Observed annual time series (blue) of mean North Atlantic (NA) total column water (TCW; kg). NA TCW is the monthly mean TCW for a given year, sampled at the month and location of every NA TC within the period 1900–2023 and averaged. Following Figure S1 of (Sparks and Toumi, 2025b).

Supplementary Tables

Table S1: Summary of the predictor variables used in the parametric TC rain model.

Model	Variable	Source	Units	Log Transform	Mean	SD
Pre-landfall	V_{max}	IBTrACS	m s^{-1}	Yes	3.82	0.23
	TCW	ERA5	kg m^{-2}	Yes	3.82	0.174
	P_m/R_m	IMERG	mm h^{-1} km^{-1}	Yes	-1.52	0.974
	P_m	IMERG	mm h^{-1}	Yes	2.46	0.568
	R_{18}	IBTrACS	km	Yes	5.23	0.401
Post-landfall	P_m	IMERG	mm h^{-1}	No	13.4	8.41
	$LandFrac_{olt}$	Natural Earth	h^{-1}	No	0.0115	0.0109
	GH_{olt}	ERA5	m h^{-1}	No	4.89	12.2

Table S2: Summary of the pre-landfall noise models.

Variable	Distribution	Shape Parameters	Location	Scale
ε_1	Lognormal	$s = 0.0781$	-6.25	6.24
ε_2	Beta	$a = 1{,}800{,}000;\ b = 5.51$	-664,000	664,000
ε_3	Weibull min	$c = 12.8$	-7.06	7.39
ε_4	Beta	$a = 17.7;\ b = 9.42$	-2.65	4.06

Table S3: Summary of the post-landfall noise models and the sampling distribution of β.

Variable	Distribution	Shape Parameters	Location	Scale
ε_5	Weibull max	$c = 4.76$	0.76	0.83
ε_6	Beta	$a = 5000;\ b = 45.6$	-30.1	30.4
β	Normal	–	0.999	0.011

Supplementary Texts

Supplementary Text S1. Description of the storyline approach.

Under global warming, trends in PI (Wehner and Kossin, 2024) and TCW (Patel and Kuttippurath, 2023; Borger et al., 2022) have been observed and are projected to continue (Pérez-Alarcón et al., 2023; Borger et al., 2022). The storyline approach to simulating the effects of global warming assumes increases of PI and TCW as the cause of TC changes. This approach is motivated by the higher confidence in the thermodynamic increases of PI (Pérez-Alarcón et al., 2023) and TCW (Borger et al., 2022), in contrast to the deep uncertainty surrounding dynamical changes projected by climate models (Knutson et al., 2020; Sobel et al., 2023; Shepherd et al., 2018). The storyline approach does not predict any changes in basin TC count or tracks.

The storyline approach assumes that the North Atlantic (NA) PI and TCW trend will follow the observed global zonal mean trend over the period 1979–2023 in the next approximately 40 years, an assumption which has been justified in Sparks and Toumi (2025b) and Sparks and Toumi (2025a). Hence, the pre-industrial and future PI and TCW climates can be estimated by an appropriate scaling of their respective trends. As in previous works, the scaling of the PI and TCW trends was determined using the global mean surface temperature anomaly record (HadCRUT5) (Morice et al., 2021) relative to the pre-industrial (1850—1900) mean. Supplementary Figure S17 shows that during the period 1979–2023, when widespread satellite observations were available, there has been an approximately linear increase in the global mean temperature. The scale parameters are determined with the following:

$$\Delta Y_{pre-ind} = \frac{T_{pre-ind} - T_{mean}}{T_{trend}} \quad (1)$$

$$\Delta Y_{2C} = \frac{T_{2C} - T_{mean}}{T_{trend}} \quad (2)$$

where $\Delta Y_{pre-ind}$ and ΔY_{2C} are the scaling parameters for the pre-industrial and future cli-

mate, respectively. T_{mean} is the mean surface temperature anomaly of 1979–2023 (+0.82 °C), T_{trend} is the regressed linear trend of the global mean surface temperature anomaly of the same period (+0.0195 °C/year), $T_{pre-ind} = 0$°C and $T_{2C} = +2$°C by design. Supplementary Figure S17 illustrates that $T_{pre-ind}$ is achieved by subtracting 43 years of T_{trend} from T_{mean} (i.e., $\Delta Y_{pre-ind} = -43$ years), while T_{2C} is achieved by adding 60 years of T_{trend} to T_{mean} (i.e., $\Delta Y_{2C} = +60$ years).

Monthly mean PI and TCW fields obtained from ERA5 were averaged for 1979–2023 to create climatological monthly mean fields, and the linear temporal trend at every grid for each month was computed. The pre-industrial and +2 °C PI were then obtained by scaling:

$$PI_{pre-ind} = PI_{mean} + \Delta Y_{pre-ind} PI_{trend} \tag{3}$$

$$PI_{2C} = PI_{mean} + \Delta Y_{2C} PI_{trend} \tag{4}$$

where PI_{mean} is the mean PI evaluated for 1979–2023 and PI_{trend} is the zonal mean linear trend over the same period at the grid point. Similarly, the pre-industrial and +2 °C TCW were obtained:

$$TCW_{pre-ind} = TCW_{mean} + \Delta Y_{pre-ind} TCW_{trend} \tag{5}$$

$$TCW_{2C} = TCW_{mean} + \Delta Y_{2C} TCW_{trend} \tag{6}$$

where TCW_{mean} is the mean TCW evaluated for 1979–2023 and TCW_{trend} is the zonal mean linear trend over the same period at the grid point. For the peak month of September, the change in the global zonally averaged September PI and TCW from pre-industrial to +2 °C is shown for the NA in Supplementary Figure S18. For PI, the largest increase occurs near 35 °N, with a relative minimum around 25 °N. By contrast, TCW exhibits its maximum increase near 25 °N, with weaker increases towards higher latitudes.

This scaling method effectively creates a storyline in which the +2 °C scenario is only driven by a change in the thermodynamics quantities, PI and TCW, which is supported by

the trend observed in the NA (see Figure S1 of Sparks and Toumi (2025b) and Supplementary Figure S19).

The control run, performed under the "present" climate of 2010, corresponds to a +1 °C scenario (Supplementary Figure S17). The same scaling method was applied to derive PI and TCW for the present climate, using a scaling factor of $\Delta Y_{1C} = +9$ years.

References

Borger C, Beirle S, Wagner T (2022) Analysis of global trends of total column water vapour from multiple years of OMI observations. Atmospheric Chemistry and Physics 22(16):10603–10621. https://doi.org/10.5194/acp-22-10603-2022, URL https://acp.copernicus.org/articles/22/10603/2022/, publisher: Copernicus GmbH

Knutson T, Camargo SJ, Chan JCL, et al (2020) Tropical Cyclones and Climate Change Assessment: Part II: Projected Response to Anthropogenic Warming. Bulletin of the American Meteorological Society https://doi.org/10.1175/BAMS-D-18-0194.1, URL https://journals.ametsoc.org/view/journals/bams/101/3/bams-d-18-0194.1.xml

Morice CP, Kennedy JJ, Rayner NA, et al (2021) An updated assessment of near-surface temperature change from 1850: The HadCRUT5 data set. Journal of Geophysical Research: Atmospheres 126(3):e2019JD032361. Publisher: Wiley Online Library

Patel VK, Kuttippurath J (2023) Increase in Tropospheric Water Vapor Amplifies Global Warming and Climate Change. Ocean-Land-Atmosphere Research 2:0015. https://doi.org/10.34133/olar.0015, URL https://spj.science.org/doi/full/10.34133/olar.0015, publisher: American Association for the Advancement of Science

Pérez-Alarcón A, Fernández-Alvarez JC, Coll-Hidalgo P (2023) Global Increase of the Intensity of Tropical Cyclones under Global Warming Based on their Maximum Potential

Intensity and CMIP6 Models. Environmental Processes 10(2):36. https://doi.org/10.1007/s40710-023-00649-4, URL https://doi.org/10.1007/s40710-023-00649-4

Shepherd TG, Boyd E, Calel RA, et al (2018) Storylines: an alternative approach to representing uncertainty in physical aspects of climate change. Climatic Change 151(3):555–571. https://doi.org/10.1007/s10584-018-2317-9, URL https://doi.org/10.1007/s10584-018-2317-9

Sobel AH, Lee CY, Bowen SG, et al (2023) Near-term tropical cyclone risk and coupled Earth system model biases. Proceedings of the National Academy of Sciences 120(33):e2209631120. https://doi.org/10.1073/pnas.2209631120, URL https://www.pnas.org/doi/abs/10.1073/pnas.2209631120

Sparks N, Toumi R (2025a) Climate change attribution of Typhoon Haiyan with the Imperial College Storm Model. Atmospheric Science Letters 26(1):e1285. https://doi.org/10.1002/asl.1285, URL https://onlinelibrary.wiley.com/doi/abs/10.1002/asl.1285, _eprint: https://rmets.onlinelibrary.wiley.com/doi/pdf/10.1002/asl.1285

Sparks NJ, Toumi R (2025b) The impact of global warming on U.S. hurricane landfall: A storyline approach. Environmental Research Letters 20(11). https://doi.org/10.1088/1748-9326/ae0956, URL http://iopscience.iop.org/article/10.1088/1748-9326/ae0956

Wehner MF, Kossin JP (2024) The growing inadequacy of an open-ended Saffir–Simpson hurricane wind scale in a warming world. Proceedings of the National Academy of Sciences 121(7):e2308901121. https://doi.org/10.1073/pnas.2308901121, URL https://www.pnas.org/doi/10.1073/pnas.2308901121, publisher: Proceedings of the National Academy of Sciences

Wright DB, Knutson TR, Smith JA (2015) Regional climate model projections of rainfall from U.S. landfalling tropical cyclones. Climate Dynamics 45(11):3365–

3379. https://doi.org/10.1007/s00382-015-2544-y, URL https://doi.org/10.1007/s00382-015-2544-y
